# Generation of surrogate brain maps preserving spatial autocorrelation through random rotation of geometric eigenmodes

**DOI:** 10.1162/IMAG.a.71

**Published:** 2025-07-16

**Authors:** Nikitas C. Koussis, James C. Pang, Richa Phogat, Jayson Jeganathan, Bryan Paton, Alex Fornito, P.A. Robinson, Bratislav Misic, Michael Breakspear

**Affiliations:** Neuromodulation Program, Hunter Medical Research Institute, New Lambton Heights, New South Wales, Australia; Mark Hughes Foundation Centre for Brain Cancer Research, College of Health, Medicine and Wellbeing, University of Newcastle, Callaghan, New South Wales, Australia; School of Psychological Sciences, Turner Institute for Brain and Mental Health, and Monash Biomedical Imaging, Monash University, Clayton, Victoria, Australia; School of Psychological Sciences, College of Engineering, Science, and the Environment, University of Newcastle, Callaghan, New South Wales, Australia; School of Medicine and Public Health, College of Health, Medicine, and Wellbeing, University of Newcastle, Callaghan, New South Wales, Australia; School of Physics, University of Sydney, Camperdown, New South Wales, Australia; Network Neuroscience Lab, Montréal Neurological Institute, McGill University, Montréal, Québec, Canada

**Keywords:** statistical brain mapping, methods, software and analysis, non-parametric inference, cortical and subcortical mapping

## Abstract

The brain expresses activity in complex spatiotemporal patterns, reflecting the influence of spatially distributed cytoarchitectural, biochemical, and genetic properties. The correspondence between these different “brain maps” is a topic of substantial interest. However, these maps possess intrinsic smoothness (spatial autocorrelation, SA) which can inflate spurious cross-correlations, leading to false positive associations. Identifying true associations requires knowledge about the distribution of correlations that arise by chance in the presence of SA. This null distribution can be generated from an ensemble of surrogate brain maps that preserve the intrinsic SA but break the correlations between maps. The present work introduces the “eigenstrapping” method, which performs a spectral decomposition of brain maps, such as fMRI activation patterns, expressed on cortical and subcortical surfaces, using geometric eigenmodes, and then randomly rotating these modes to produce SA-preserving surrogate brain maps. It is shown that these surrogates appropriately represent the null distribution of chance pairwise correlations, with expected false positive control superior to current state-of-the-art procedures. Eigenstrapping is fast, eschews the need for parametric assumptions about the nature of a map’s SA, and works with maps defined on smooth surfaces with a boundary, such as a single cortical hemisphere when the medial wall has been removed. Moreover, eigenstrapping generalizes to broader classes of null models than existing techniques, offering a unified approach for inference on cortical and subcortical maps, spatiotemporal processes, and complex patterns possessing higher-order correlations.

## 1 Introduction

Interest in spatial patterns of cortical activity, cellular and microstructural composition, molecular architecture, and network connectivity of the brain has surged in recent years (Arnatkeviciute et al., 2022; Fornito et al., 2019; Hansen et al., 2021, 2022; Markello et al., 2022; Rubinov & Sporns, 2010). An important challenge in this field is to measure the similarity between two or more such “brain maps” while excluding spurious relationships arising from chance (Friston et al., 2000; Worsley et al., 1998). Correlations between different maps may reflect, for example, the influence of spatially patterned gene expression on cytoarchitecture or neuronal activity, hence motivating further mechanistic investigation (Fornito et al., 2019; Liu et al., 2023). However, cortical regions that are close together tend to possess similar features, the causes of which may be biological (such as a gradual change in gene expression) or methodological (due to the spatial smoothing that is applied in the analyses of most imaging modalities). These effects combine to endow brain maps with a spatial autocorrelation (SA) that typically has an extent of tens of mm (Alexander-Bloch et al., 2018; Burt et al., 2020; Markello et al., 2022; Viladomat et al., 2014). The presence of such within-map correlations reduces the true degrees of freedom when testing for pairwise associations between maps, hence amplifying spurious associations (Afyouni et al., 2019). Null hypotheses of the correspondence between maps, that is, the distribution of “chance” in map-to-map correlations, need to preserve SA to control Type I error (Burt et al., 2020; Markello & Misic, 2021). This is not a trivial undertaking in the presence of complex statistical dependencies within and between maps (Váša & Mišić, 2022).

There are several methods that can generate surrogate maps that maintain SA while randomizing the association between maps, hence providing suitable “null models.” Most of these null models fall into two broad classes: (1) direct spatial permutation, commonly known as the “Spin Test” (Alexander-Bloch et al., 2018; Baum et al., 2020; Cornblath et al., 2020; Kuhn, 1955; Váša et al., 2018), whereby maps in the neocortex are projected onto a sphere, randomly rotated, then projected back to the cortical surface; and (2) parameterized spatial randomization, such as “Brain Surrogate Maps with Autocorrelated Spatial Heterogeneity” (BrainSMASH), whereby surrogate maps are drawn from a random (Gaussian) process and smoothed to closely match the empirical SA with parametric models that approximate the original statistical structure (Burt et al., 2018, 2020). However, both classes have drawbacks: the Spin Test provides incomplete coverage of the cortex because it rotates missing data in the medial wall (i.e., vertices within the subcortex and anatomically inferior to the cingulate) onto the map (see Fig. 5 for a demonstration). In addition, the Spin Test has thus far not been extended to volumetric maps, precluding its use in the subcortex. The Spin Test also preserves the original spatial relationships between all points, only rotating them to different locations. This form of randomization yields a restricted null space with an assumption that no higher-order spatial structure exists within the original map. Higher-order spatial effects occur frequently in biological systems, including neural processes in visual cortex, reflecting the complex statistical dependences in natural scenes (Hermundstad et al., 2014; Puckett et al., 2020; Tesileanu et al., 2020; Victor et al., 2015). Estimating the null space to identify these more complex spatial effects requires a randomization of higher-order statistical dependencies. Conversely, generating spatial nulls with spatial parametric techniques such as BrainSMASH (Burt et al., 2020) requires extensive parameter optimization and is computationally intensive (Markello & Misic, 2021). Moreover, these methods rest upon parametric assumptions about the SA, drawing randomness from stationary Gaussian processes. Cortical activity frequently violates these assumptions, exhibiting long-tailed statistics and nonlinear spatiotemporal properties (Deco et al., 2011; Freyer et al., 2009, 2011; Heitmann & Breakspear, 2018; Tognoli & Kelso, 2014; van den Heuvel et al., 2012). At high levels of SA, both of these methods fail tests of Type I error, with false positive rates 2–10 times higher than expected (Markello & Misic, 2021). This inflation can be particularly problematic for inference on smooth, lower-resolution maps, such as those generated with brain transcriptomics or positron emission tomography.

To improve on the current methods to generate null models, we turn to geometric basis sets for cortical surfaces. These basis sets—known as geometric eigenmodes—support the decomposition of complex spatial patterns expressed on a cortical surface from coarse to fine wavelengths. Geometry constrains the behavior of many complex systems, including the brain, where it influences large-scale dynamics such as standing and traveling waves (Greicius et al., 2009; Muller et al., 2014, 2016; Nunez & Cutillo, 1995; Roberts et al., 2019). Geometric eigenmodes have increasingly been used to model and describe these diverse aspects of brain activity and structure (Chen et al., 2022; Gabay et al., 2018; Gabay & Robinson, 2017; Henderson et al., 2022; Nunez & Srinivasan, 2006; Robinson et al., 2016; Tokariev et al., 2019). Geometric eigenmodes are essentially spherical harmonics generalized to non-spherical surfaces and can be derived by application of the Laplace–Beltrami operator (LBO) (Gabay & Robinson, 2017). Notably, for the present purposes, the LBO projects spatial data into an orthogonal subspace similar to a Fourier decomposition of a one- or two-dimensional system on a regular grid (Bullmore et al., 2004; Laird et al., 2004; Theiler et al., 1992). This allows constrained random rotation of the basis set without disrupting the SA, akin to random rotation of the phase of a Fourier decomposition, a well-established and widely used surrogate method in time series analysis (Theiler et al., 1992). Appropriate eigenmode randomization can thus yield a geometric surrogate map preserving the SA of the original data while randomizing the location and higher-order properties of the map. In this sense, where the Spin Test is analogous to cyclic permutation in brain maps, eigenmode randomization is analogous to Fourier phase randomization in these maps.

Here, we introduce *eigenstrapping*, a method of generating random brain maps with preserved SA for null hypothesis testing. By constrained rotation of geometric eigenmodes, eigenstrapping provides a method to perform rigorous statistical inference of cortical and subcortical associations and surface or volumetric maps for a broad range of research questions. We show that eigenstrapping has expected false positive rate (FPR) control across a wide range of SA, low computational burden, generalizability to a broad class of spatial processes, use in both cortical and subcortical maps, and applicability to complex spatial and spatiotemporal processes (Alexander-Bloch et al., 2018). We provide an open-source Python-based package that is deployable to commonly utilized neuroimaging formats (Koussis & Systems Neuroscience Group, 2024).

## 2 Methods

### 2.1 Statistical testing of spatial associations between brain maps

Let two vectors y(x) and z(x) denote brain maps on a discretized cortical surface x with *N* vertices. The sample correlation coefficient ${\hat{\rho }}_{yz}$ measures the strength and direction of the linear relationship between these two maps *y* and *z* across the surface,

$$\begin{array}{c} {\hat{\rho }}_{yz}=\frac{\sum_{i=1}^{N}\left[\left(y\left(x_{i}\right)-\bar{y}\right)\left(z\left(x_{i}\right)-\bar{z}\right)\right]}{{\hat{\sigma }}_{y}{\hat{\sigma }}_{z}}, \end{array}$$ (1)

where $\bar{y}$ and $\bar{z}$ are the average values of *y* and *z* over ***x***; ${\hat{\sigma }}_{y}$ and ${\hat{\sigma }}_{z}$ are the corresponding (sample) standard deviations, and $x_{i}$ denotes the *i-*th vertex. The null hypothesis ${ℋ}_{0}$ that the association between the two maps is random implies that the empirical test statistic ${\hat{\rho }}_{yz}$ is drawn from a null distribution centered at zero, that is, $E({\hat{\rho }}_{yz})=0$ . If the maps possess no autocorrelation—that is, they are spatially white noise—then the variance of the test statistic ${\hat{\rho }}_{yz}$ under the null is simply a function of the number of vertices as given by

$$\begin{array}{c} var\left({\hat{\rho }}_{yz}\right)=\frac{1}{N}. \end{array}$$ (2)

However, if the maps y(x) and z(x) possess SA, the amount of information they carry is less than if they were independent—hence the effective sample size is $\hat{N}$ is smaller than N. This adjustment inflates the size of the distribution of the test statistic under the null (Brown & Rutemiller, 1977; Clifford et al., 1989). In the special case where the smoothness in each map arises from a simple Gaussian process with isotropic autocorrelation, then the variance of the test statistic under the null obeys a parametric solution which incorporates the autocorrelation functions $r_{y}$ and $r_{z}$ (see Supplementary Information S1). In this setting, the statistical significance of a linear correlation between two specific maps can be inferred via a standard parametric test. However, empirical brain maps—cortical activity, PET ligand binding, microstructural composition, molecular architecture, etc.—typically possess heavy-tailed, anisotropic and/or multiscale spatial autocorrelations, violating these assumptions (Eklund et al., 2016; Roberts et al., 2015).

When the nature of the SA is complex or unknown, an alternative is to generate surrogate brain maps $y^{\prime }\left(\text{x}\right)$ and $z^{\prime }\left(\text{x}\right)$ that preserve the SA of the empirical maps y(x) and z(x) but do not possess any specific cross-dependence. Accordingly, the variance of the surrogate cross-correlation approximates the variance of the original cross-correlation—$var({\hat{\rho }}_{y^{\prime }z^{\prime }})≅var({\hat{\rho }}_{yz})$ , but the mean is centered at zero $\langle {\hat{\rho }}_{y^{\prime }z^{\prime }}\;=0\rangle$ where the expectation is taken over a large number of individual surrogate realizations. This procedure provides an estimate of the true distribution for the test statistic under the null hypothesis and thus allows inference on the observed cross-correlation ${\hat{\rho }}_{yz}$ against this null distribution. Eigenstrapping is a nonparametric method for achieving this through rotation of cortical eigenmodes.

### 2.2 Eigenmode decomposition of cortical maps

An eigenmode decomposition on a discretized surface x with *N* vertices yields *N*-1 orthogonal eigenmodes (when excluding the uniform mode as in the current study) which can be ordered by their corresponding eigenvalues. These modes are the solution to the Helmholtz equation of the Laplace–Beltrami operator (LBO) on the surface ***x***,

$$\begin{array}{c} \Delta \psi_{\eta }\left(\text{x}\right)=-\lambda_{\eta }\psi_{\eta }\left(\text{x}\right), \end{array}$$ (3)

where $\Psi ={\left\{\psi_{\eta }\right\}}_{\eta =0}^{\infty }$ are the eigenmodes, ${\left\{\lambda_{\eta }\right\}}_{\eta =0}^{\infty }$ are the corresponding eigenvalues, and η=0,1,2,… index the modes. These modes provide a spectral basis decomposition of a brain map or other spatial pattern y(x) from fine to coarse wavelengths (i.e., spatial frequencies) (Gabay & Robinson, 2017; Pang et al., 2023; Robinson et al., 2016).

In the case of spherical surfaces, the eigenmodes are called spherical harmonics and occur in groups Λ of modes with identical (degenerate) eigenvalues. For a sphere with *N* uniformly spaced vertices, there are a maximum of $\Lambda_{max}=\sqrt{N/2}-1$ groups each with μ=2(Λ+1) modes. In this setting, each harmonic group describes a set of orthogonal spatial patterns expressed on the sphere that vary over the same wavelength. The folds, gyri, and non-spherical distortions of the cortical geometry perturb this structure, but the mean eigenvalue separation between adjacent groups of modes is approximately preserved, particularly at spatial scales relevant for whole brain maps, allowing one to use similar groupings (Robinson et al., 2016; see Supplementary Information S2).

**Fig. 1.**
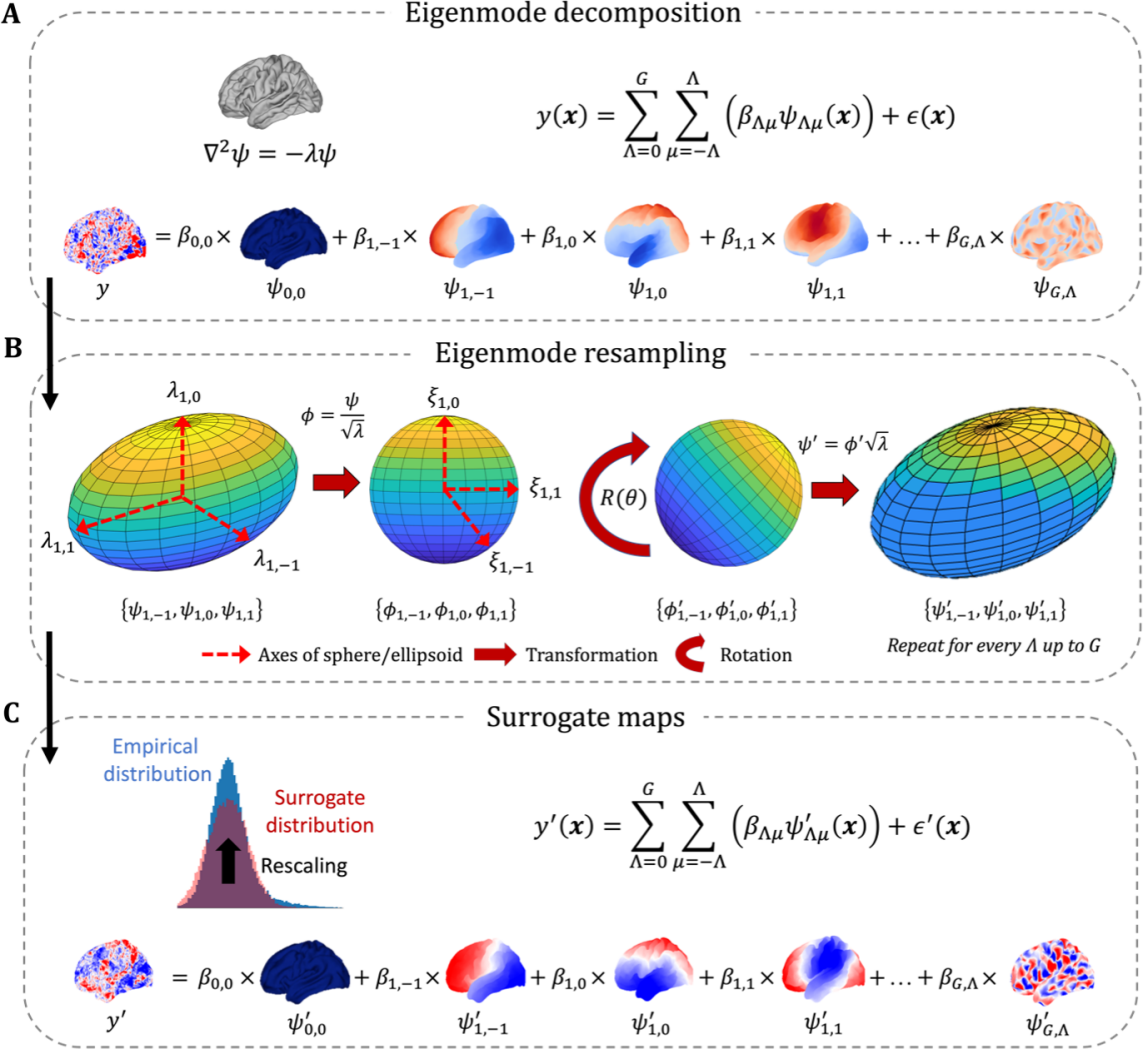
The *eigenstrapping* method to generate surrogates that preserve spatial autocorrelation. (A) *Eigenmode decomposition*: coefficients $\beta_{\Lambda \mu }$ are derived from the generalized linear model (GLM; Eq. 5). A total number of modes is chosen such that the residual error in the GLM is negligible. (B) *Eigenmode rotation*: Eigenmodes are partitioned into eigengroups Λ (of size n=2Λ+1 ) and normalized by their eigenvalues $\lambda_{\Lambda \mu }$ to yield spherical eigenmodes $\phi_{\Lambda \mu }$ with identical eigenvalues $\xi_{\Lambda \mu }$ . This is analogous to transformation from an *n*-dimensional ellipsoid with axes $\lambda_{\Lambda \mu }$ to an *n*-dimensional sphere with axes $\xi_{\Lambda \mu }$ . The equality of ξ (i.e., degeneracy; Supplementary Fig. S1) permits rotation of $\phi_{\Lambda \mu }$ by a random rotation matrix R(θ), resulting in rotated spherical eigenmodes $\phi_{\Lambda \mu }^{\prime }$. These modes are multiplied by $\sqrt{\lambda_{\Lambda \mu }}$ to project them back to the ellipsoid, resulting in rotated modes $\psi_{\Lambda \mu }^{\prime }$ in groups Λ. (C) *Surrogate maps*: The GLM with original coefficients β is multiplied by rotated modes $\psi^{\prime }$ across all Λμ yielding a surrogate brainmap $y^{\prime }$ . An optional amplitude adjustment step (*rescaling*; see Supplementary Information S4) is applied to the reconstructed data (in red; original data in blue). Residuals can be permuted and added back into the resulting surrogate map $y^{\prime }$ (see Supplementary Information S3).

Formally, an empirical brain map y(x) on a discrete surface x is decomposed into a linear combination of geometric eigenmodes,

$$\begin{array}{c} y\left(\text{x}\right)=\sum_{\Lambda =0}^{G}\sum_{\mu =-\Lambda }^{\Lambda }\left(\beta_{\Lambda \mu }\psi_{\Lambda \mu }\left(\text{x}\right)\right)+\in \left(\text{x}\right), \end{array}$$ (4)

where *G* is the total number of groups of modes and there are 2Λ+1 modes in each group. $\beta_{\Lambda \mu }$ is the linear coefficient (weighting) of mode $\psi_{\Lambda \mu }$ in group Λ with eigenvalue $\lambda_{\Lambda \mu }$ (see Fig. 1A).

Coefficients β are estimated from empirical data by taking the inner product of the modes ψ with the brain maps *y* on the surface ***x*** (Hilbert, 1985; Robinson et al., 2021),

$$\begin{array}{c} \beta_{\Lambda \mu }=\langle y\left(\text{x}\right),\;\psi_{\Lambda \mu }\left(\text{x}\right)\rangle . \end{array}$$ (5)

The residual error 0(x) in Eq. (4) decreases in amplitude as the number of modes used in the decomposition increases, vanishing if the decomposition is complete, that is, if the full complement of N−1 modes is used. Geometric eigenmodes can be adapted to surfaces defined within a closed boundary such as a single cortical hemisphere when the non-physiological medial wall has been removed. We employ Neumann boundary conditions—that is, the derivative of the cortical surface normal to the boundary is set to zero when the eigenmodes are estimated (Supplementary Information S2). The cortical map y(x) is then masked to remove the excluded medial wall before the inner product (4) is estimated (Appendix A). This reflects the common approach taken in neuroimaging, which is that the medial wall should be considered as a boundary and data within the medial wall are not physiological (Fischl et al., 1999). The Neumann boundary condition was chosen rather than the Dirichlet condition (fixing the value along the boundary rather than the derivative) which introduces a discrete, hard edge between the map and the medial wall (Reuter et al., 2009; Wachinger et al., 2015).

### 2.3 Random rotation of eigengroups

Individual modes within a group Λ are orthogonal by virtue of the relative orientation, while the groups themselves are also orthogonal due to their differing wavelengths. We use this orthogonality between groups to resample modes without disrupting the spatial spectra and hence SA of the map *y*. Spherical harmonics within groups possess identical spatial frequencies and are rotationally invariant. Geometric eigenmodes adapt to the folds and undulations of the cortical surface. As a consequence, they are not rotationally invariant and modes within a group possess similar but not identical spatial frequencies (see Supplementary Information S2). To rotate geometric eigenmodes within groups, it is thus necessary to normalize their eigenvalues to have equal value, equivalent to mapping the modes onto an *n*-dimensional sphere z,

$$\begin{array}{c} \Phi_{\Lambda }\left(\text{z}\right)=\Psi_{\Lambda }\left(\text{x}\right)⊙\kappa_{\Lambda }, \end{array}$$ (6)

where $\Phi_{\Lambda }$ is the equivalent spherical representation of $\Psi_{\Lambda }$ (both in matrix form), and $\kappa_{\Lambda }$ is a vector of $\kappa_{\Lambda i}=\lambda_{\Lambda i}^{-1/2}$ . Eq. (6) represents the element-wise scaling [Hadamard product (⊙)] of $\Psi_{\Lambda }$ by the vector $\kappa_{\Lambda }$, the (element-wise) inverse square root of $\lambda_{\Lambda }$, which is the vector of geometric eigenvalues with group number Λ. The number of modes in a group and hence the dimension of the sphere remain n=2Λ+1 (Supplementary Table S1 and Supplementary Fig. S2).

As a result of the normalization Eq. (6), all spherical modes within a group have identical eigenvalues denoted $\xi_{\Lambda 1}=\xi_{\Lambda 2}=\ldots \;\xi_{\Lambda n}.$ To perform eigenstrapping, groups of spherical modes are rotated by taking the matrix dot product with a random rotation matrix $\text{R}\left(\theta_{\Lambda }\right)$ (Blaser & Fryzlewicz, 2016),

$$\begin{array}{c} {\Phi^{\prime }}_{\Lambda }\left(\text{z}\right)=\Phi_{\Lambda }\left(\text{z}\right)R\left(\theta_{\Lambda }\right), \end{array}$$ (7)

where the prime denotes rotation by a random angle θ and Λ is the group number. Note that Eq. (7) describes a matrix multiplication of the spherical eigengroup by random rotation matrix R. This group-based rotation ensures spherical modes within groups retain their orthogonality. This process is repeated with an independent random rotation applied to each group, breaking the original angular alignment of modes across different groups. In contrast to the Spin Test, rotations are applied group wise to separate spherical eigenspaces, increasing the number of realizations and disrupting higher-order correlations, while still constraining randomization to the smoothness of the original map.

Rotated spherical modes $\phi_{\Lambda \mu }^{\prime }\left(\text{z}\right)\;$ are then mapped back to the original geometry, yielding rotated geometric eigenmodes,

$$\begin{array}{c} \Psi_{\Lambda }^{\prime }\left(x\right)=\Phi_{\Lambda }^{\prime }\left(\text{z}\right)⊙\zeta_{\Lambda }, \end{array}$$ (8)

where $\zeta_{\Lambda i}=\lambda_{\Lambda i}^{1/2}$ , renormalizing the operation of Eq. (6).

A surrogate brain map $y^{\prime }\left(\text{x}\right)$ is then obtained from these rotated eigenmodes,

$$\begin{array}{c} y^{\prime }\left(\text{x}\right)\sum_{\Lambda =0}^{G}\sum_{\mu =-\Lambda }^{\Lambda }\left(\beta_{\Lambda \mu }\psi_{\Lambda \mu }^{\prime }\left(\text{x}\right)\right)+\in^{\prime }\left(\text{x}\right), \end{array}$$ (9)

where $\beta_{\Lambda \mu }$ are the same coefficients from Eq. (4), while the surrogate error term $\in^{\prime }\left(\text{x}\right)$ can be derived from simple random permutation of the error term ∈(x) from Eq. (4) if required (see Section 2.4). The resampling procedure is illustrated in Figure 1B for the first non-zero eigengroup, Λ=1 .

Note that the surrogate brain map is reconstructed as a sum of geometric modes each with random phase alignment, yielding statistical independence between each mode. According to the Central Limit Theorem, the sum of many independent, random variables converges to a Gaussian distribution. Accordingly, and in common with wavelet and Fourier resampling methods (Breakspear et al., 2003), surrogate brain maps will have an amplitude distribution that converges to a Gaussian distribution, regardless of the amplitude distribution of the empirical map (Schreiber & Schmitz, 1996). This potentially causes a mismatch between the original and surrogate. To preserve the amplitude distribution of the empirical data, an optional amplitude-adjustment step can be performed (Fig. 1C, top left; Supplementary Information S4).

### 2.4 Depth of decomposition and treatment of residuals

The eigenmode decomposition Eq. (1) is complete for a total of *N-*1 modes when performed on a spatial mesh with *N* vertices. Accordingly, the residual term ∈(x) in Eq. (4) approaches zero (within numerical accuracy). However, for a highly resolved cortical mesh (e.g., 32,492 vertex points for *fs-LR-32k*), a complete representation carries a substantial computational burden. In practice, the SA of neuroimaging data is typically far coarser than current finely resolved cortical meshes and the error term becomes small for substantially fewer eigenmodes than *N-*1. Consequently, most of the variance of a cortical map can be reconstructed with relatively few eigenmodes. Rotation of an incomplete eigenmode decomposition (up to g≪G groups) represents a computationally parsimonious way of resampling cortical maps. In this case, the surrogate error term $\in^{\prime }\left(\text{x}\right)$ can be derived from simple random permutation of the error term ∈(x) from Eq. (4). Alternatively, the error term can be set to zero as the associated loss of variance in the surrogate data can be recovered by application of the amplitude adjustment step (see Supplementary Information S4 and Supplementary Figs. S5–7).

**Fig. 2.**
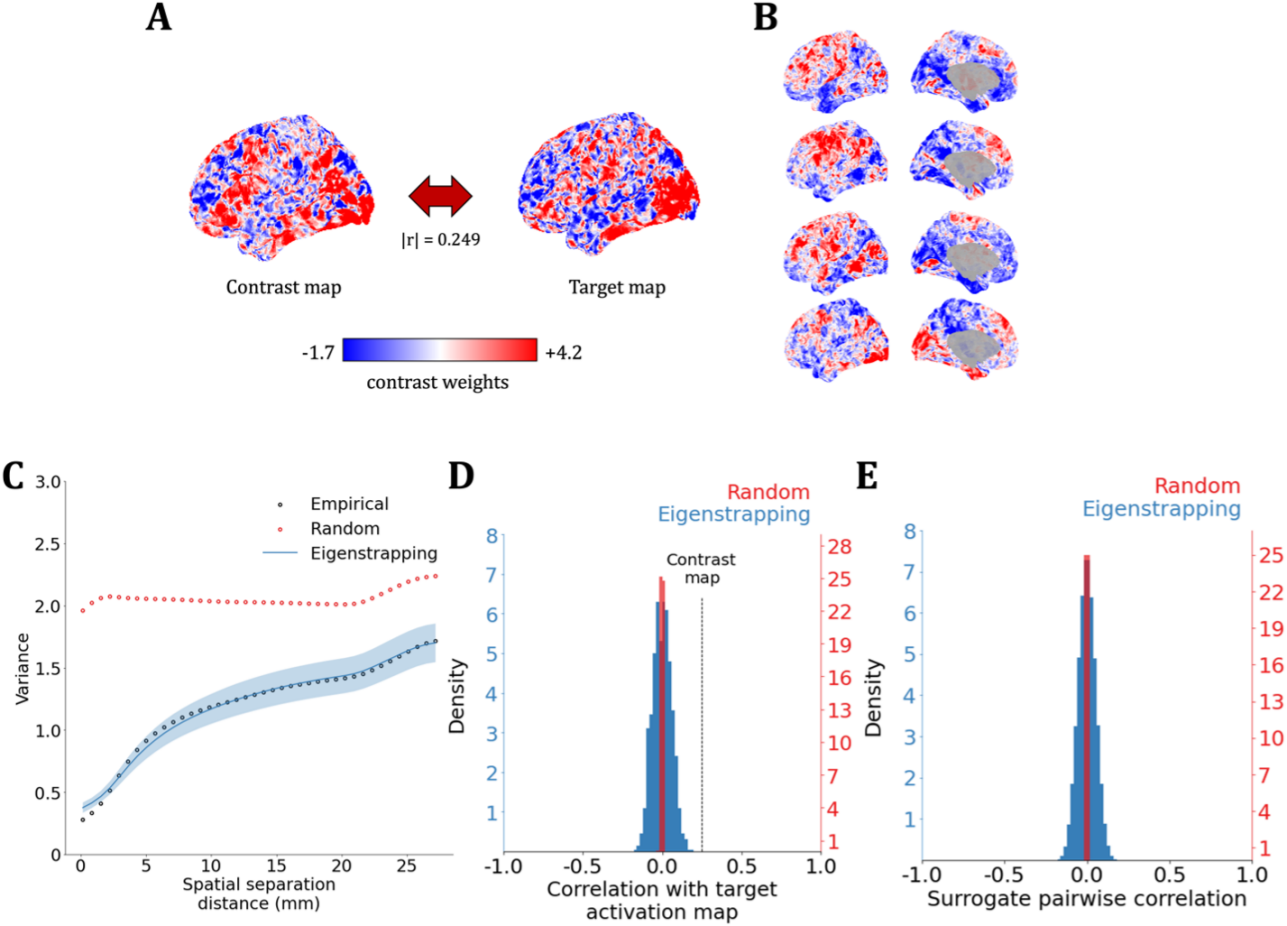
Statistical properties of eigenstrapped surrogates of fMRI data. (A) An example HCP task contrast map (*Contrast map*; left) was correlated with a target contrast map (*Target map*; right) from the same participant in another task condition at |r| = 0.249. Each map is colored by task contrast weight. (B) Four example eigenstrapped surrogates of the contrast map (panel A, left). (C) The variogram from 0 to 30 mm spatial separation with the average of 1,000 SA-naïve surrogates (*Random*; red circles), and the average and standard deviation of 1,000 eigenstrapping surrogates (*Eigenstrapping*; blue line and shading, respectively) against the contrast map (*Empirical*). (D) Correlation of SA-naïve (red) and eigenstrapping (blue) surrogates with the target fMRI map. The correlation of the target map to the contrast map |r| = 0.249 is shown with the dashed black line. In this case, the correlation lies outside the null distribution and is thus considered statistically significant. (E) Pairwise correlation of SA-naïve (red) and eigenstrapping (blue) surrogates.

For initial analyses (Figs. 1 and 2, on the *fs-LR-32k* pial surface), we computed the first 6,000 modes to test the algorithm, corresponding to ~18.5% of the complete set of surface modes. For analysis on simulated maps (performed on the *fsaverage5* pial surface), we computed the first 2,500 modes, corresponding to ~ 2.5% of all surface modes.

### 2.5 HCP data

We used task fMRI data from the Human Connectome Project (HCP) (Glasser et al., 2013, 2016). Activation maps were downloaded in CIFTI format in *fs-LR-32k* standard space from 255 unrelated subjects for the *social cognition task*, *motor task*, *gambling task*, *working memory task*, *language task*, *emotion task*, and *relational task*, accessing 47 task contrasts in total (see Supplementary Information S5).

### 2.6 Gaussian random fields

The statistical properties of eigenstrapping were benchmarked using parametric simulations of Gaussian random fields (GRFs), adopting a previous approach (Markello & Misic, 2021). Simulated brain maps were derived by generating pairs of 3D multivariate Gaussian distributions with random correlation. These ensembles of pairs were generated with seven different levels of SA across by modifying the slope of the pair’s power spectral density (α= 0.0–3.0 in increments of 0.5, where 0.0 indicates random Gaussian noise). The pairs of GRFs were projected to the fsaverage5 cortical mesh using FreeSurfer *mri_vol2surf*. The medial wall was removed (i.e., set to NaN), mimicking empirical maps. At each level of SA, we generated 1,000 pairs, resulting in 7,000 total pairs of maps with random correlations centered at ρ  = 0.0 (Supplementary Information S6).

### 2.7 Null comparison: Spin Test

The Spin Test (a version of a classical spatial permutation test) randomizes the alignment between two cortical surface maps through rotation by a random angle (Alexander-Bloch et al., 2018), and is useful for computing SA-corrected *p*-values when making statistical inferences on dense cortical brain maps. To compare eigenstrapping with the null brain maps generated using the Spin Test, we used the Python implementation of the method in the *neuromaps* toolbox (Markello et al., 2022). Any statistics drawn from Spin Test generated maps excluded the rotated medial wall (as NaNs) as the standard implementation. As the spin test can only produce null maps of the cortical surface, we used another method for comparison of both cortical surface measures and volumetric measures, namely, the BrainSMASH method, outlined below.

### 2.8 Null comparison: BrainSMASH

The Brain Surrogate Maps with Autocorrelated Spatial Heterogeneity (BrainSMASH) method uses geostatistical methods to derive randomized brain maps that replicate the empirical map’s SA (Burt et al., 2020). The steps that the BrainSMASH tool uses are as follows: (1) randomly permute the values in a target brain map and (2) smooth and rescale the permuted map to recover the SA structure of the target brain map. This is performed through rescaling of values in several spatial levels of linear fits of Gaussian, exponential, or logarithmic distributions. To generate null brain maps using the BrainSMASH method, we used the Python implementation from https://github.com/murraylab/brainsmash. The dense sampling algorithm (*brainsmash.mapgen.Sampled*) was used for all analyses in this study. As the method allows for different fits to the variogram depending on parameters given, optimized parameters were chosen based on visual assessment of best fit to the original variogram.

### 2.9 Cortico-subcortical functional connectivity patterns

We used resting-state functional connectivity patterns (“gradients”) to examine the capacity of eigenstrapping to identify cortical–subcortical effects. Resting-state data from the HCP were sampled on tetrahedral meshes (thalamus, hippocampus, and the striatum—consisting of the caudate, putamen, and nucleus accumbens areas). These structures were generated from binarized images of 25% probability thresholds of the Harvard–Oxford subcortical atlas of each region (Desikan et al., 2006; Frazier et al., 2005; Goldstein et al., 2007; Makris et al., 2006).

Cortico-subcortical functional gradients were derived from diffusion map embedding of HCP resting-state fMRI (see Supplementary Information S8). The first non-zero gradient for each subcortical structure (corresponding to the second eigenvector in Eq. S15) was derived from the Laplacian of the group-averaged resting-state functional connectivity matrix of HCP data in MNI152 space. Eigenmodes were derived on the tetrahedral mesh, resampled to volumetric space, then rotated, yielding SA-preserving surrogate subcortical maps (Fig. 5).

### 2.10 Local texture patterns

A variety of spatial images and processes, such as natural scenes, possess complex spatial features that cannot be fully captured using standard first-order statistics (two-point correlations, or spatial autocorrelation) (Maddess et al., 2004). We refer to these higher order effects as textures to emphasize the complex arrangement of spatial features above low-level smoothness. Complex texture features (local ternary patterns; LTPs) (Gupta et al., 2010) were analyzed to quantify the effect of eigenstrapping on the presence of these features in natural scenes. By discretizing an image into three values (-1, 0, 1), LTPs can be used to detect complex, textural effects such as facial features (see Results and Supplementary Information S10).

## 3 Results

### 3.1 Statistical properties of surrogate maps generated from rotated eigenmodes

We first generated eigenstrapped surrogate maps from task-evoked fMRI data on the *fs-LR-32k* surface of 255 unrelated healthy individuals from the Human Connectome Project (Van Essen et al., 2013) (HCP; *emotion*, see Supplementary Table S2 for a list of tasks). This was compared with surrogates generated from SA-naïve random permutation of vertices. An example HCP participant’s target map (HCP; *gambling*) is compared with an example contrast map (HCP; *emotion* map) in Figure 2A with Pearson’s correlation r  = 0.249. These two maps illustrate the problem of inferring the presence or absence of a weak-to-moderate correlation between two reasonably smooth maps. To demonstrate its face validity in smooth brain maps, Figure 2 presents a comparison of eigenstrapping to a SA-naïve permutation.

Example surrogate maps using an eigenmode decomposition with 6,000 modes visually capture the smoothness of the original data (Fig. 2B). Quantifying the SA of these maps using the variogram, a measure of local smoothness (Burt et al., 2020) shows that eigenstrapping (Fig. 2C, blue) preserves the empirical SA to very small spatial separations (<1 mm). In contrast, SA-naïve random permutations whiten (pairwise decorrelate) the surrogates, producing relatively flat SA (Fig. 2C, red). An ensemble of 1,000 eigenstrapped surrogate maps exhibits a broad, zero-centered distribution of correlations with a target empirical map (Fig. 2D, blue; *gambling*), hence yielding a wider distribution than SA-naïve random permutations (Fig. 2D, red). Notably, eigenstrapped surrogate maps are on average uncorrelated with each other, yielding a broad, zero-centered pairwise-correlation distribution (Fig. 2E), demonstrating the face validity of the method.

### 3.3 Control of false positives in simulated brain maps

**Fig. 3.**
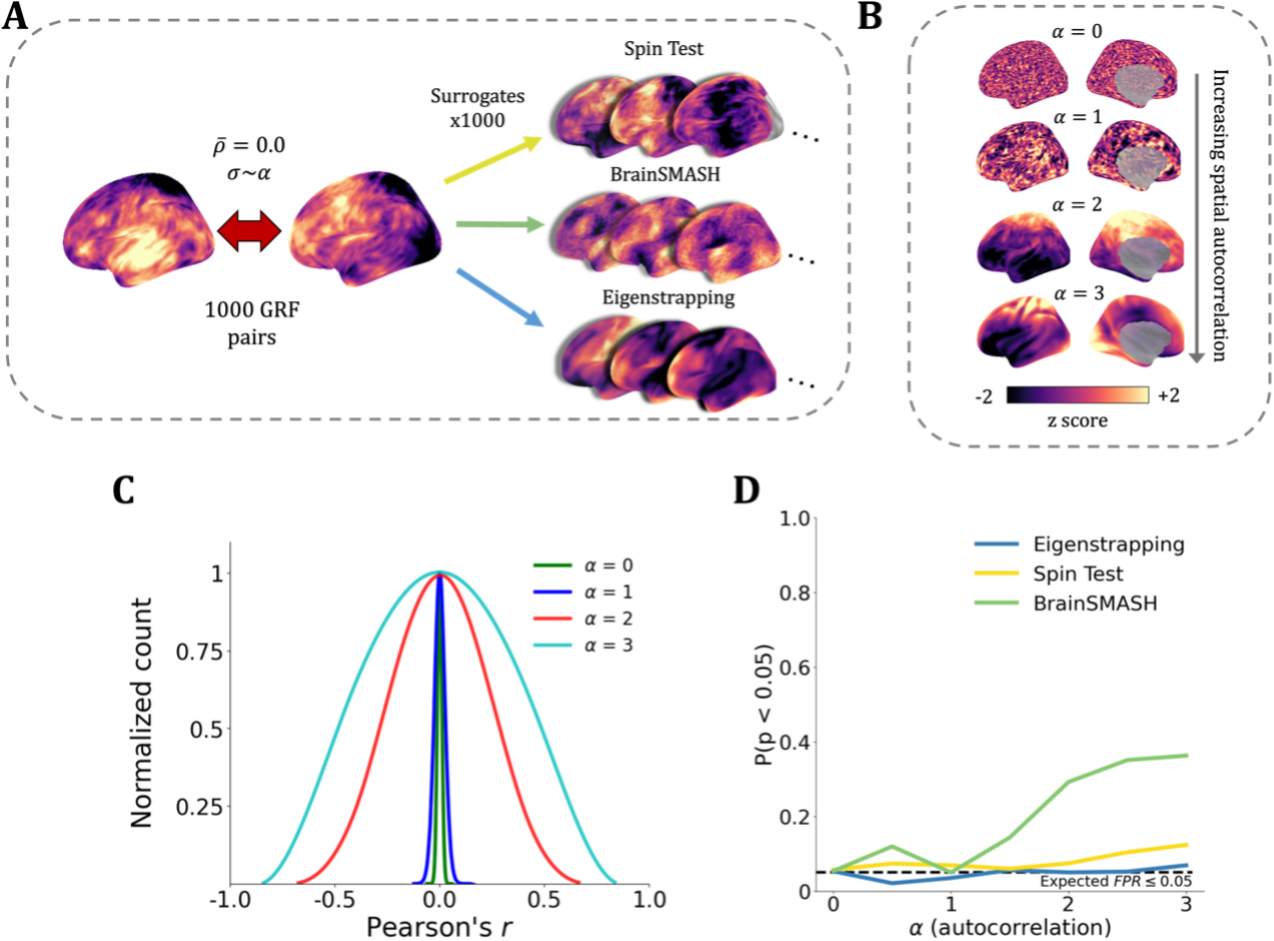
Eigenstrapping control of false positives. (A) Gaussian random fields (GRFs) with varying SA were used to generate pairs of cortical maps with random cross-correlation. The average correlation between two maps was zero, while the variance σ of the correlations increases proportionally with α. In total, 1,000 surrogates were generated per GRF pair. A GRF pair is plotted at α = 2 alongside Spin Test (yellow), BrainSMASH (green), and eigenstrapping (blue) exemplar surrogates. (B) GRFs are plotted with α increasing from 0.0 to 3.0. (C) Average null distributions of eigenstrapping for different levels of SA, normalized between 0 and 1. (D) Each line indicates false positive rate (FPR) of null method as a function of α (eigenstrapping: blue; Spin Test: yellow; BrainSMASH: green). The black dashed line corresponds to an expected FPR of ≤5%.

We next tested the efficacy of eigenstrapping in controlling Type I error, benchmarked against a ground truth from simulated brain maps. Simulated maps were generated with Gaussian random fields (GRFs) that have parametrically varying SA (Cohen, 1998; Yura & Hanson, 2011) with smoothness parameter α (Fig. 3; see Methods and Supplementary Information S6). We simulated pairs of GRF maps with random cross-correlations that were on average centered at zero, and a cortical resolution of 10,242 vertices in the *fsaverage5* standard space. Smoothness was tuned from α = 0.0 (no SA) to α = 3.0 (high SA) in steps of 0.5 with 1,000 pairs of GRFs generated at each step (Fig. 3A, B). This procedure yielded 7,000 total pairs with pairwise cross-correlations centered at ρ  = 0.0. As with previous uses of this procedure (Burt et al., 2020; Markello & Misic, 2021), the variance of the cross-correlation distribution widens as a function of α (Fig. 3C), increasing the chances of false positives.

We used eigenstrapping to derive a *p*-value for the cross-correlation of the simulated pairs of cortical maps. In total, 1,000 surrogate maps were derived from one map in each GRF pair using eigenstrapping (Fig. 3A, blue) with fixed number of modes. The eigenstrapped surrogates and the other map in the GRF pair were correlated, forming a correlation distribution across α levels (Fig. 3C). The choice of modes was derived by a heuristic that truncated the number of modes to the minimum number of whole groups that had a characteristic FWHM greater or equal to the average FWHM of the GRF pairs (see Supplementary Information S2 and Supplementary Figs. S3–4). These ranged from N = 2,500 to 2,000 at α = 0.0–1.0, 850 to 550 at α = 1.5, 50 to 15 at α = 2.0, 25 to 8 at α = 2.5, and 8 to 3 at α = 3.0. The resulting correlation distributions were then used to estimate the two-tailed *p*-value for the original correlation of the GRF pair. As SA increases, the distribution of the correlation of eigenstrapped surrogates and GRF pairs (the estimate of the null distribution) widens (Fig. 3C).

We next benchmarked the false positive rate (FPR) of eigenstrapping against those of the Spin Test and BrainSMASH. Since the correlations between the randomly paired GRFs are zero centered, the FPR should be equal to or below the chosen statistical alpha—that is, ≤5% FPR at *p* < 0.05. Eigenstrapping yields an FPR near or below the expected 5% for α = 0.0–2.5 (Fig. 3D in blue), with 5.3% (α = 0.0), 2.1% (α = 0.5), 3.5% (α = 1.0), 5.6% (α = 1.5), 5.0% (α = 2.0), 5.2% (α = 2.5), and 5.2% (α = 3.0). For the same test, the Spin Test yields higher than expected FPR across all SA regimes, ranging from 5.7% at α = 0.0 (Fig. 3D in yellow), increasing to 6.8% (α = 1.0), 7.4% (α = 2.0), to a maximum of 12.3% (α = 3.0). The BrainSMASH method shows higher FPR than both the Spin Test and eigenstrapping, reaching 29.2% at α = 2.0 and 36.3% at α = 3.0 (Fig. 3D in green). Note that the FPR increases for all methods when the generated GRF maps are smoother than those seen empirically (i.e., for α > 3.0).

We further quantify the SA-preserving property of eigenstrapping by calculating Moran’s *I*, a measure of global SA^50^, for each GRF and surrogate map (Supplementary Figs. S8 and S9). In contrast to the variogram, which captures two-point correlations as a function of distance, Moran’s *I* provides a single composite summary of SA (Anselin, 1995; Burt et al., 2020). Eigenstrapping preserves Moran’s *I* for all levels of smoothness α (see Supplementary Information S7 and Supplementary Figs. S10–11).

**Fig. 4.**
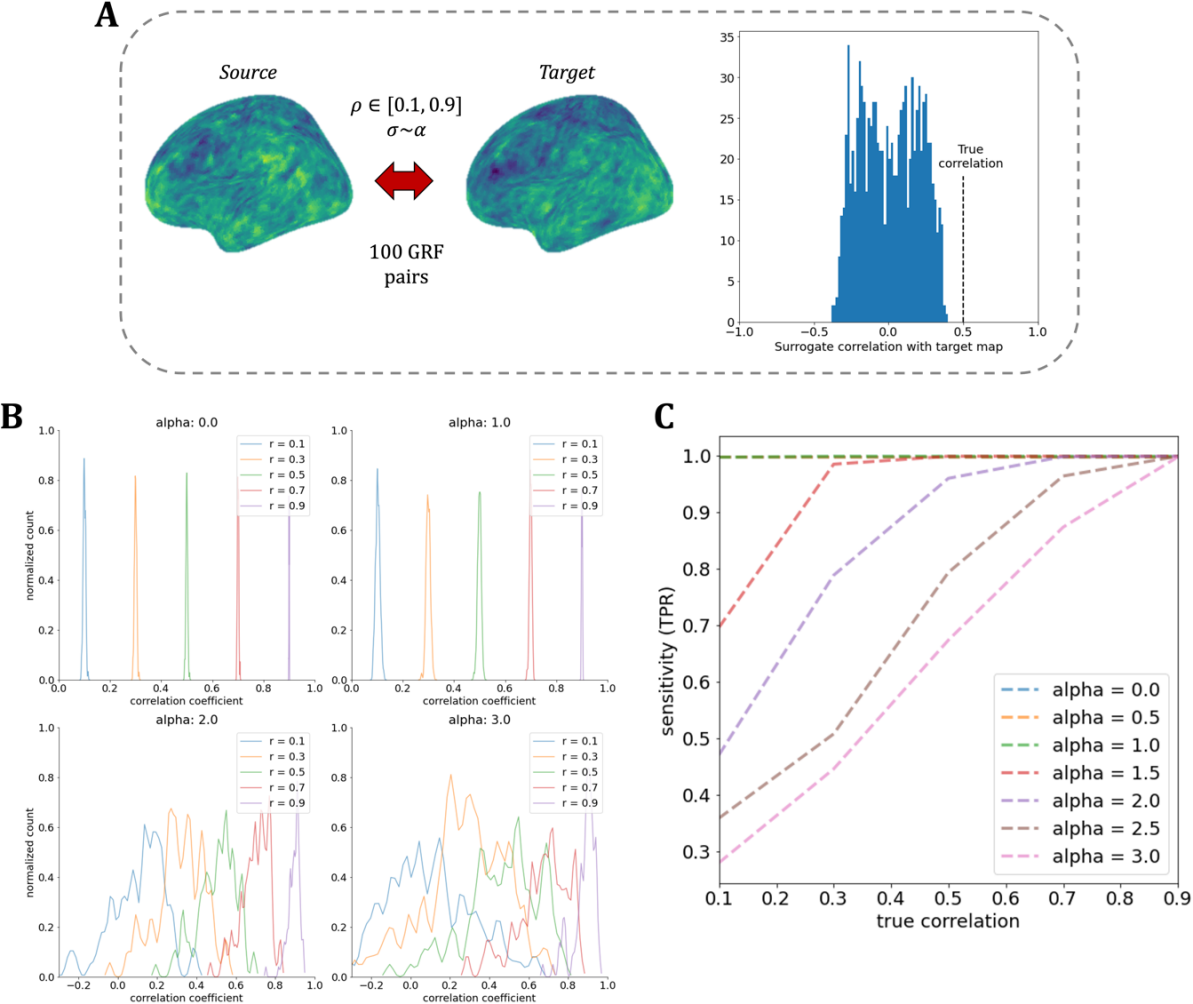
Eigenstrapping sensitivity. (A) 100 GRF pairs were simulated with fixed correlations ρ  = 0.1–0.9 in steps of 0.2. For each *source–target* GRF pair, 1,000 eigenstrapping surrogates were generated (with *N* modes derived by the FWHM of the *source* map, as defined in Supplementary Information S2) and correlated with the *target* map to produce a non-parametric null distribution. An example pair and surrogate correlation distribution of α  = 1.5 and ρ  = 0.5 is shown. (B) Correlations of *source* and *target* GRF pairs centered at each ρ widen as α increases. (C) Sensitivity (true positive rate; TPR) as a function of correlation level ρ. Dashed colored lines represent different α (α = 0.0: blue; α = 0.5: orange; α = 1.0: green; α = 1.5: red; α = 2.0: purple; α = 2.5: brown; α = 3.0: magenta).

We tested the sensitivity of eigenstrapping by generating 100 pairs of GRFs with fixed correlations ρ  = 0.1–0.9 (in steps of 0.2; Fig. 4A). The true positive rate (TPR) for each correlation ρ was then derived for different levels of smoothness (α = 0.0–3.0; Fig. 4B) by correlating 1,000 surrogate maps of the source map with the target map for each of the correlated GRF pairs. Eigenstrapping shows high sensitivity for noisy maps (α = 0.0, 0.5, 1.0), with TPR close to unity across all correlations (Fig. 4C). For moderate smoothness (α = 1.5, 2.0), the TPR remains close to 1.0 for strong correlations, but falls for increasingly smooth maps (α = 2.5, 3.0) as the true correlation falls within the null. This is consistent with predictions from parametric results, which show that the variance of the test statistic under the null becomes increasingly wide with smoother data such that true correlations become difficult to detect (Supplementary Information S1).

### 3.4 Null hypothesis testing of associations between empirical brain maps

**Fig. 5.**
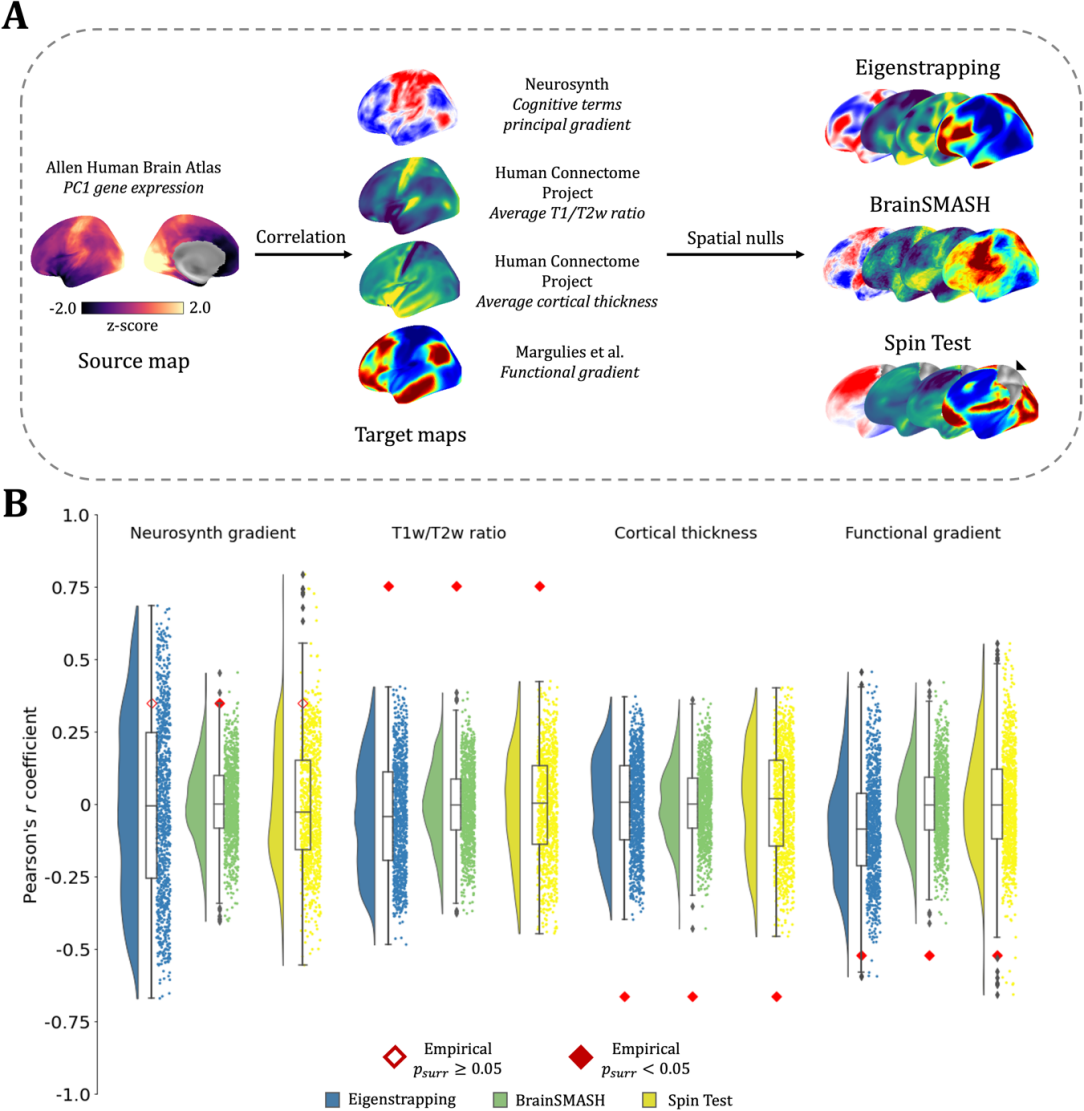
Null hypothesis testing of associations between empirical brain maps. (A) Examining the association of the first principal gradient (PC1) of cortical gene expression (left) with four example target maps (center). The exemplar surrogates per method are plotted on the left hemisphere of the inflated *fsaverage* surface. Inference on these associations was performed using three SA-preserving surrogate methods (eigenstrapping: blue; BrainSMASH: green; Spin Test: yellow) (B) Correlations with source brain map (first principal gradient of gene expression) of target brain maps (*Neurosynth gradient, T1w/T2w ratio, cortical thickness,* and *functional gradient*) in red; surrogate correlations to source map plotted with rainclouds (M. Allen et al., 2021) (eigenstrapping: blue; BrainSMASH: green; Spin Test: yellow). Empirical correlations of source/target pairs are given by red-bordered (non-significant, $p_{surr}$ ≥ 0.05) or red-filled (significant, $p_{surr}$ < 0.05) diamonds. All *p*-values are family-wise error corrected (Miller, 2012).

A primary goal of using surrogate brain maps is to identify significant associations between effects expressed on the cortical mantle—where a ground truth is lacking—such as the correlation between the spatial pattern of a gene’s expression and spatially distributed activation patterns or cortical morphology (Fornito et al., 2019; Markello et al., 2022; Vos de Wael et al., 2020). We next explored associations between the first principal component (PC1) of gene expression (Markello et al., 2021) (Fig. 5A, left) with well-validated surface maps (Fig. 4A, middle) of function (the principal gradient of cognitive terms from functional activation studies; *Neurosynth*) (Poldrack et al., 2011; Yarkoni et al., 2011); structure (the average ratio of T1-weighted to T2-weighted MRI) (Glasser et al., 2013; Van Essen et al., 2013); morphology (average cortical thickness) (Glasser et al., 2013; Van Essen et al., 2013); and intrinsic functional connectivity (the first principal component of resting-state functional connectivity) (Margulies et al., 2016). We performed inference on these associations using surrogates derived from eigenstrapped surrogates (blue) and compared the results with the BrainSMASH (green) and Spin Test (yellow) methods. Empirical correlations were *z*-scored to quantify the relative effect size and statistical significance of each null (Fig. 5B).

The correlations of each of these maps with gene expression vary considerably in magnitude and sign (see red diamonds in Fig. 5B). The association of the cognitive gradient with the gene expression map is the weakest (Fig. 5B, left; *Neurosynth gradient*). Notably, the null is only rejected for the BrainSMASH test (z = 2.55, p = 0.008), whereas the nulls derived from the two other methods possess wider tails which enclose the empirical correlation (eigenstrapping z  = 1.14, p = 0.39; Spin test z = 1.71; p = 0.062). All other associations are statistically significant (i.e., *p* < 0.05) when using any of the nulls, although the correlation distributions are consistently narrowest for the BrainSMASH test, yielding larger *z*-statistics for the T1w/T2w ratio (eigenstrapping z = 4.18, BrainSMASH z = 5.69, Spin Test z = 4.18), cortical thickness (eigenstrapping z = -4.10, BrainSMASH z = -5.34, Spin Test z = -3.59), and the functional gradient (eigenstrapping z = -2.42; BrainSMASH z = -3.86, Spin Test z = -2.90).

The narrower tails of the BrainSMASH method are notable and could be due to a whitening effect on the SA, which is evident in the noisier visual appearance of these nulls (Fig. 5A, middle; Supplementary Fig. S12A). Although the SA is preserved to the width of the kernel, a lack of smoothness is present at larger separation distances (i.e., the variogram is flatter; Supplementary Fig. S12B). This issue does not occur with the eigenstrapped nulls (Supplementary Fig. S12C). Very long-wavelength SA (captured by the eigenspectrum) is preserved with eigenstrapping but degraded by the BrainSMASH test (Supplementary Fig. S12D, E). Although the Spin Test preserves the SA, the rotation of the medial wall is evident—the black marker on the Spin Test surrogate (Fig. 5A: *Spin-permuted*, rightmost brain map) indicates the non-data (NaNs) from the medial wall that are rotated onto the cortical surface. This issue is avoided by eigenstrapping as eigenmodes are only derived within the closed, bounded cortical surface (see Methods and Supplementary Information S9).

### 3.5 Generating subcortical surrogate maps

**Fig. 6.**
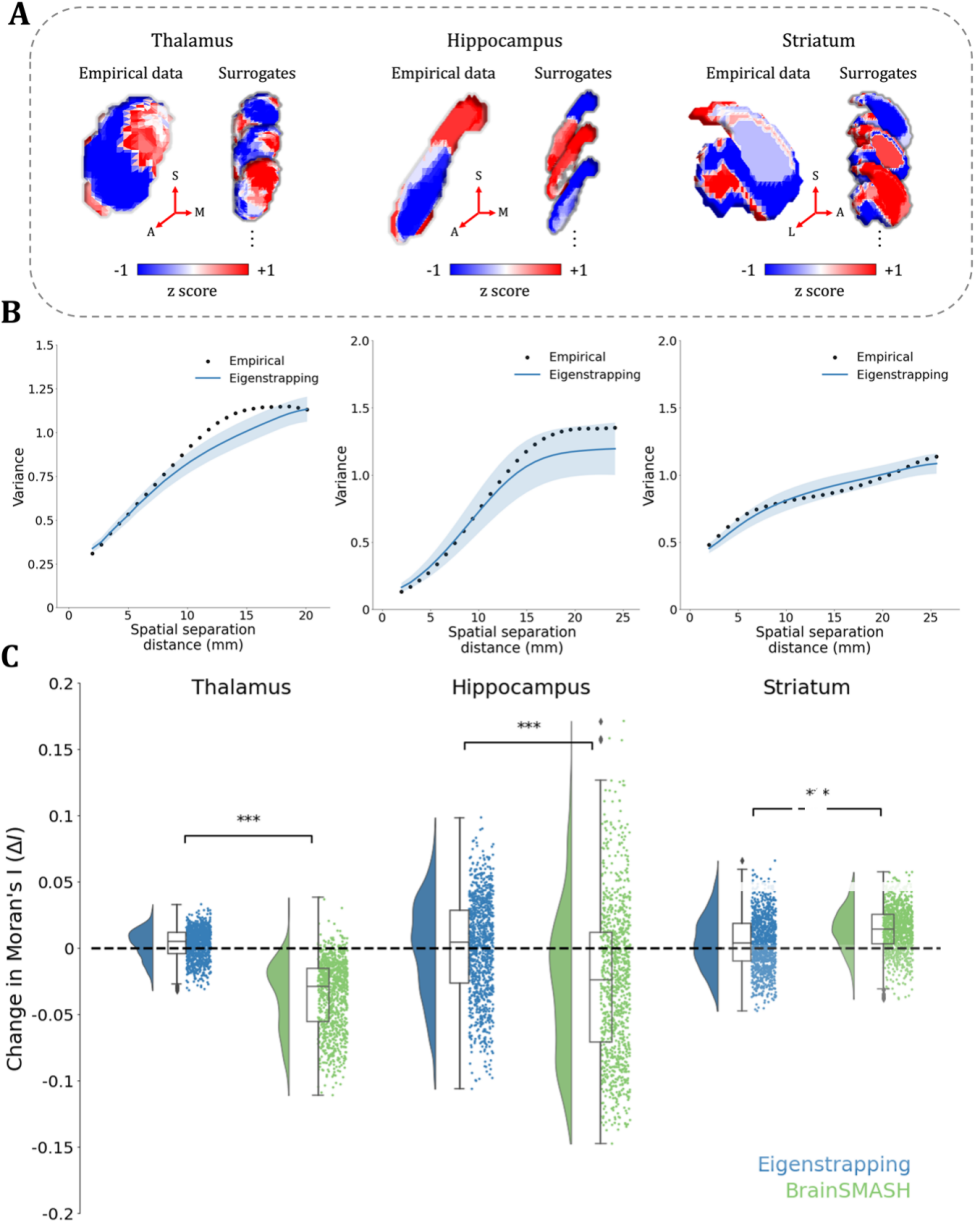
Subcortical surrogate maps with spatial autocorrelation. (A) Cortico-subcortical connectivity gradients (*empirical data*) in left thalamus (left), hippocampus (middle), and striatum (right), and three example surrogates generated using eigenstrapping. Number of modes used were 700, 100, and 300 for thalamus, hippocampus, and striatum, respectively, and all surrogates had amplitude adjustment applied. Thalamus surrogates also had residuals permuted. Axes of subcortical projections are given by red arrows: S: superior; A: anterior; M: medial; L: lateral. (B) Variograms of subcortical principal gradients (black) and 1,000 surrogates (blue) across the three subcortical structures. (C) Change in Moran’s *I* (Δ*I*) for difference in SA within principal gradients. Rainclouds of 1,000 surrogates of eigenstrapping (blue) shown against BrainSMASH (green) for each subcortical structure. Black bars denote *T*-tests performed between each null method ΔI . Stars correspond to significance level of two-sided *p*-values of *T*-tests: ***: *p* < 0.005; **: *p* < 0.01; *: *p* < 0.05; n.s.: *p* ≥ 0.05.

Characterizing subcortical activity and cortical–subcortical interactions is of substantial current interest (Borne et al., 2023; Buckner et al., 2011; Guell et al., 2018; Haak et al., 2018; Margulies et al., 2016; Marquand et al., 2017; Tian et al., 2020; Vos de Wael et al., 2020). We extended eigenstrapping to volumetric data to enable significance testing of associations between and within subcortical structures. As a demonstration, we constructed tetrahedral meshes of three subcortical structures (thalamus, hippocampus, and striatum; see Methods) and applied eigenstrapping to these discretized surfaces. The process for generation of eigenstrapping surrogate maps in subcortical and volumetric spaces is identical to the process for cortical surfaces once a mesh has been derived. In brief, subcortical (tetrahedral) geometric eigenmodes are transformed to the spherical representation and randomly rotated, then transformed back, producing subcortical surrogate maps with matched SA as in Eq. (2). As an example, we derived maps of cortico-subcortical associations (known as “functional connectivity gradients”, see Supplementary Information S8), which capture the principal variations of functional connectivity between subcortical and cortical voxels. For present purposes, this method yields smoothly varying patterns projected onto thalamus, hippocampus, and striatum, from which we derive eigenstrapped surrogates (Fig. 6A).

The application of eigenstrapping to these structures generates subcortical surrogates that preserve the variety of SA in these data (Fig. 6B). Eigenstrapping preserves empirical SA (change in Moran’s *I*; ΔI ) more accurately than BrainSMASH with optimized parameters (Fig. 6C). Specifically, the changes in Moran’s *I* were significantly lower in eigenstrapping surrogates compared with BrainSMASH surrogates across all subcortical structures (*thalamus*: Student’s *T-*statistic (*T*) = 42.24, *p* < 0.0005, *degrees of freedom* (*d.f.*) = 1998; *hippocampus*: *T* = 12.27, *p* < 0.0005, *d.f.* = 1998; *striatum*: *T* = -11.46, *p* < 0.0005, *d.f.* = 1998). The skewness of the distributions of thalamus and hippocampus (Fig. 6C: left and middle distributions, respectively) may reflect the anisotropy of smoothness in these structures. For example, they may possess higher variability of SA along their long axes. The effect may also be due to finite sample size effects as each of these structures comprise fewer vertices (≤1,500), compared with the striatum (>2,500 vertices). Notably, both eigenstrapping and BrainSMASH demonstrate skewness in Moran’s I suggesting this is a phenomenon arising from the surfaces themselves, and not due to the specific resampling method. Note that the Spin Test cannot currently generate surrogates of volumetric maps, so it could not be compared with the eigenstrapping and BrainSMASH results in these subcortical structures.

### 3.6 Higher-order spatial correlations and complex textural features

While SA captures the linear, two-point smoothness of a pattern, many spatial maps possess higher order correlations, with ternary (three-point) and quaternary (four-point) relationships that cannot be predicted from knowledge of standard (two-point) correlations. These complex textures arise in systems showing accumulative and thermochemical processes such as soils (Campos Mantovanelli et al., 2021), alloys (Berman & Allison, 2021), and gene enrichment in plants (Flores-Núñez et al., 2020). Ternary and quaternary effects are also present in natural scenes (Maddess et al., 2004), where they are central to human visual perception (Hermundstad et al., 2014; Tesileanu et al., 2020; Victor et al., 2015) and associated responses in visual cortex (Puckett et al., 2020).

**Fig. 7.**
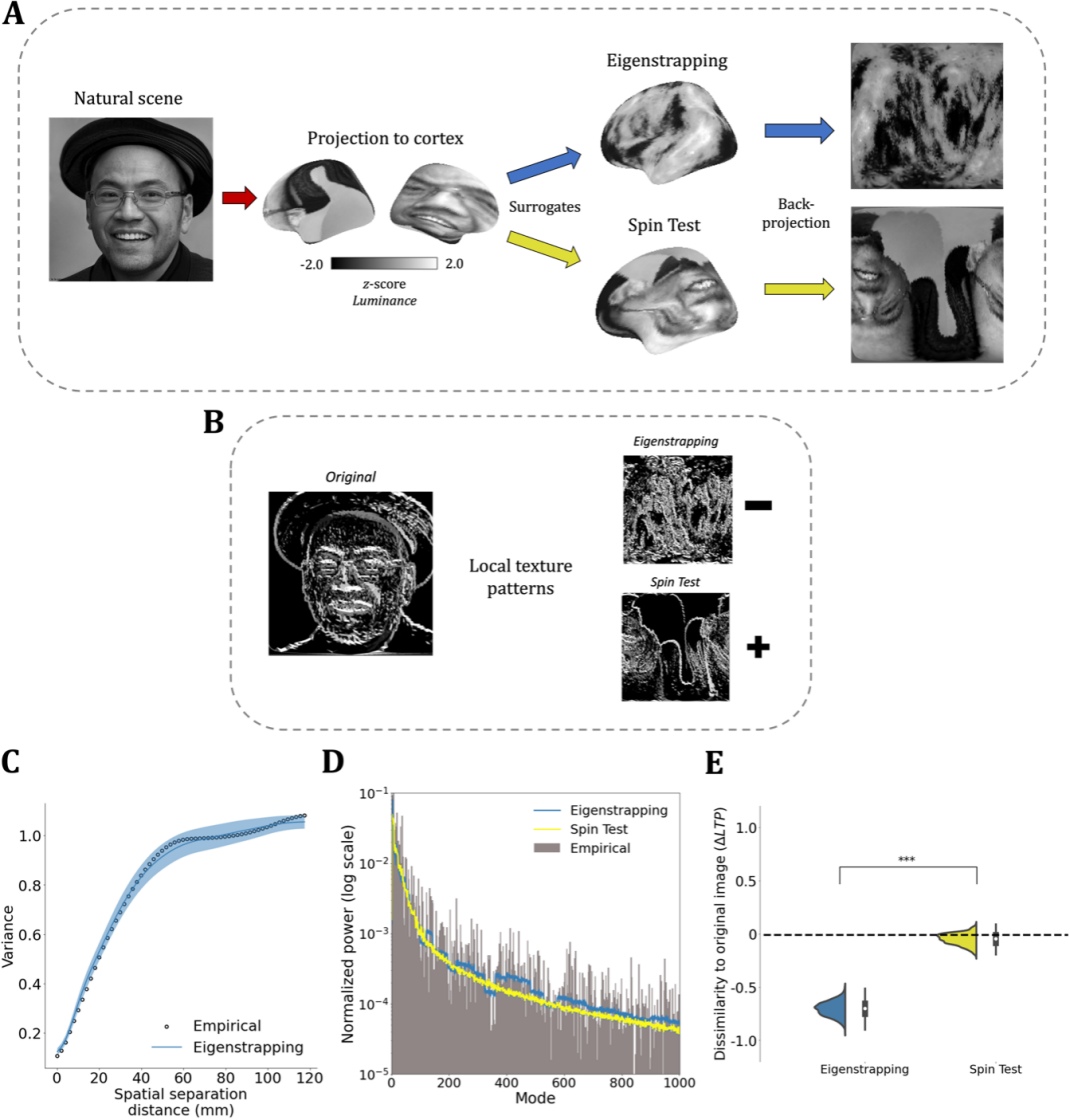
Eigenstrapping randomizes complex textural features in a natural scene. (A) A grayscale image of a natural scene (an artificially generated face) projected to the cortex. In total, 1,000 surrogates were generated from eigenstrapping (blue) or the Spin Test (yellow). Luminance values are *z*-scored and kept constant throughout the analysis. Surrogates are then projected back to the square grid using nearest-neighbor interpolation. (B) Grid-projected images are discretized using local texture patterns (local ternary patterns, LTP; Gupta et al., 2010), which classify values (-1: black, 0: gray, 1: white) based on the similarity of a local neighborhood to a central pixel. (C) The variogram of the eigenstrapping surrogates follows the empirical curve from very fine to coarse spatial scales. (D) The average modal power spectra of the surrogates are nearly identical (Pearson’s *r* = 0.949) and reproduce the empirical power spectrum (gray) (Pearson’s *r* = 0.62 and 0.50 for eigenstrapping and Spin Test, respectively). (E) The proportion change in local ternary patterns $\left(\Delta LTP=\frac{empirical\;\sum LTP-surrogate\;\;\sum LTP}{empirical\sum LTP}\right)$ for each surrogate method.

Many effects expressed on the cortex arise from complex biophysical processes. It is hence possible that many cortical maps, such as ocular dominance stripes, possess complex textural properties. Establishing their presence requires a surrogate method that preserves low order (binary) correlations but randomizes higher-order (ternary, quaternary, *etc.*) correlations. As a proof of principle, we project a human face, a canonical multiscale natural scene, to the cortex (Fig. 7A). The Spin Test rotates this cortical map and distorts but does not disrupt the complex textural relationships when projected back to the grid (Fig. 7A, bottom). It thus provides an insufficiently deep randomization of the original map. In contrast, eigenstrapping preserves two-point correlations (first-order smoothness) but visually randomizes these more complex cross-scale properties (Fig. 7A, top) due to the randomization of effects across independently rotated scales (eigengroups).

To test this effect more formally, we discretized the images using local ternary patterns (LTP (Gupta et al., 2010)), which classify values based on the similarity of a local neighborhood to a central pixel (Fig. 7B, see Supplementary Information S10). Both eigenstrapping and the Spin Test preserve the variogram and the eigenspectrum of the face (Fig. 7C, D) but only eigenstrapped surrogates disrupt these textural properties (Fig. 7E). The difference between the methods’ proportion ΔLTP is substantial (T = 237.81, p < 0.0005, d.f. = 1998). Eigenstrapping thus presents a unique method to generate a null distribution for identifying complex textural properties in brain maps.

**Fig. 8.**
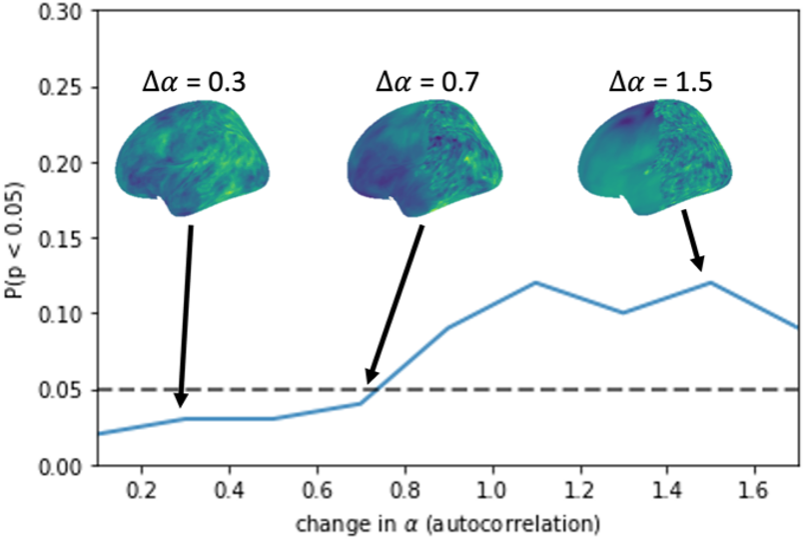
Eigenstrapping control of false positives in spatially heterogeneous maps. FPR shown in blue for increasing Δα . Example heterogeneous maps with high SA front halves and low SA rear halves. *Left surface*: front α = 2.0; rear α = 1.7; Δα = 0.3. *Center surface:* front α = 2.2; rear α = 1.5; Δα = 0.7. *Right surface:* α = 2.6, rear α = 1.1; Δα = 1.5.

The presence of spatial heterogeneity in the smoothness of brain maps can inflate the FPR of spatial null methods (Leech et al., 2024). We undertook a proof-of-principle robustness study of the performance of eigenstrapping in the presence of spatial heterogeneity, by generating GRF maps with increasingly stark heterogeneities in the anterior versus posterior half of a single hemisphere. Specifically, we generated 100 *high SA* GRF pairs with α = 1.9–2.7 in steps of 0.1 and 100 *low SA* pairs at α = 1.1–1.8 in steps of 0.1. For each pair, we constructed a heterogeneous map by cutting the front half vertices of the *low SA* map and replacing them with the corresponding vertices of the *high SA* map, resulting in 100 pairs with a change in SA or Δα = 0.1–1.7 (in steps of 0.2). For example, for Δα = 0.1, the rear half of the map possesses α = 1.8 and the front half of the map possesses α = 1.9. For Δα = 0.3, the rear half was α = 1.7 and the front half of the map was α = 2.0 (Fig. 8).

These analyses show that eigenstrapping brain maps with heterogeneous SA yields appropriate control of false positives even when the difference in SA is moderately large (Δα = 0.7, corresponding to a difference in average FWHM of >50 mm). Above this value, the FPR increases above its nominal value of 0.05 (Fig. 8).

## 4 Discussion

We present a method to generate surrogate brain maps by resampling geometric basis sets. Eigenstrapping yields a very large number of surrogate realizations while closely preserving the complex spatial smoothness of the original data. These additional realizations explore a deeper null space than other methods, generating surrogate maps that preserve two-point correlations but randomize more complex textural properties. By imposing a boundary, eigenstrapping also avoids the “medial wall problem” and can be extended from cortical meshes to three-dimensional subcortical grids. Eigenstrapping preserves the full spatial power spectrum, preserving spatial correlations well beyond the spatial smoothing kernels that lie at the core of the BrainSMASH method. For this reason, eigenstrapping preserves the Moran’s local *I* statistic more faithfully than the BrainSMASH test and does not require parametric assumptions or extensive parameter tuning, with eigenstrapping only having one free parameter (the number of modes used for decomposition; see Supplementary Fig. S13). Improvements over the current methods are summarized in Table 1. We provide an open-access Python package, which implements eigenstrapping for surface and volumetric maps (Koussis & Systems Neuroscience Group, 2023). The various advantages of eigenstrapping over existing methods are noted in Table 1.

**Table 1.** Comparison of eigenstrapping to other null models.

Metric	Surface	Eigenstrapping	Spin Test	BrainSMASH
*Computation time*	Cortical^1^	10^3^ seconds (**<10^2^ seconds with permutations**)	**10^2^ seconds**	10^4^ seconds
	Volumetric^2^	**10^1^ seconds**	*Cannot perform volumetric nulls*	10^3^ seconds
*Spatial autocorrelation*	Cortical^3^	**≤10^-5^**	**≤10^-5^**	**≤10^-5^**
	Volumetric^4^	**0.0037**	*Cannot perform volumetric nulls*	0.0041
*Maximum false positive rate*	Cortical	**5.3%**	13.5%	36.3%

Feature		Eigenstrapping	Spin Test	BrainSMASH
*Disruption of higher-order effects*		**✓**	**✗**	**✓**
*Parallel maps* ^5^		**✓**	**✗** ^†^	**✗**
*Morphospaces* ^6^		**✓**	**✗**	**✗**
*Cortico-subcortical connectivity*		**✓**	**✗**	**✓**

1 Average magnitude of computation time of 1,000 surrogates on *fsaverage5* surface using 1 CPU thread in log-seconds (see Supplementary Table 3 for testing computer specifications).

2 Average magnitude of computation time of 1,000 surrogates of striatum volume using 1 CPU thread in log-seconds (Supplementary Figs. S14–15).

3 Magnitude of mean change in Moran’s *I* in cortical maps at α=3 (Fig. 3).

4 Mean change in Moran’s *I* in cortico-subcortical gradients in Figure 6.

5 By fixing randomization of topology at the first volume, fMRI can be resampled at each subsequent volume, thereby examining which fluctuations arise by chance and what brain activity is significant.

6 Partial randomization of topology can occur by rotating iteratively or at specific modal wavelengths, exploring the spatial dependencies and topological morphospace of the original data.

† The rotations of the Spin Test could be fixed across fMRI volumes, but due to the sizable loss of vertices during medial wall insertion, it is less than ideal for providing a fully complete spatiotemporal 4D null.

By rotating spatial modes within groups and recomposing a map with the coefficients from the original eigenmode decomposition, eigenstrapping preserves the average amplitude of each spatial frequency, and as such is a natural extension of Fourier phase randomization (Laird et al., 2004; Theiler et al., 1992) and wavelet resampling methods (Breakspear et al., 2003, 2004; Laird et al., 2004), to spatial data on curved and folded surfaces. We show that eigenstrapping permutes higher order (ternary) properties of spatial maps, just as Fourier phase randomization permutes comparable nonlinear properties in time series data. Although it is generally accepted that the brain expresses nonlinear activity (Breakspear, 2017; Deco et al., 2011; Freyer et al., 2009, 2011; Tognoli & Kelso, 2014), the presence and putative function of nonlinear properties of brain maps is an empirical question for which we provide the inferential tools. Preserving the SA of one or both maps in a test of linear association, but degrading higher order properties, such as tertiary correlations and nonlinearities, does not impact the corresponding linear test statistic. This is demonstrated via appropriate control of the FPR (Fig. 3). Although inference regarding linear effects could be performed without destroying nonlinear and tertiary effects, the null space explored is much smaller and risks (near-)co-alignment of the original and rotated surrogate data. This is compounded by the presence of the medial wall issue for the spin test, which eigenstrapping avoids through the use of a Neumann boundary condition. The depth of randomization also allows eigenstrapping to test for more nuanced effects, such as the presence of tertiary or nonlinear effects. Although this is a nascent field in brain maps, evidence of such nonlinearities in neurophysiological data has facilitated novel insights into the nature of adaptive (Freyer et al., 2011) and pathological brain states (Breakspear et al., 2006).

Eigenstrapping lends itself to an extension to spatiotemporal data, again by importing a technique from multivariate phase randomization of time series data (Breakspear et al., 2003; Prichard & Theiler, 1994): Applying the same random rotation of each eigengroup across whole brain volumes acquired sequentially through time preserves temporal properties of each point-wise time series and spatial relationships between time series, while randomizing all other spatial properties. Excursions outside this spatiotemporal null would be informative regarding complex physiological processes, such as the presence of traveling waves (Aquino et al., 2012; Gabay et al., 2018; Muller et al., 2014, 2016; Nunez & Cutillo, 1995; Nunez & Srinivasan, 2006) and metastable dynamics (Freyer et al., 2009, 2011; Roberts et al., 2019). More broadly, any metric sensitive to time-dependent functional connectivity could be employed to detect non-trivial fluctuations in brain state, which are of substantial current interest (Aedo-Jury et al., 2020; E. A. Allen et al., 2014; Calhoun et al., 2014; Choe et al., 2017; Lindquist et al., 2014; Lurie et al., 2020; Menon & Krishnamurthy, 2019; Zalesky et al., 2014). Demonstrating these effects will be the subject of future work.

Identifying a suitable orthogonal transformation that removes the complex correlations within spatiotemporal data is key to nonparametric methods, as this allows rotation of the phases of basis functions without degrading the correlations of the original data (Bullmore et al., 2001; Nichols & Holmes, 2002). Resampling methods for null hypothesis testing of neuroimaging data have previously employed the discrete wavelet transform for this endeavor (Breakspear et al., 2004; Patel et al., 2006). However, while wavelet-based resampling methods are suitable for data on regular two-dimensional grids (such as fMRI slices) (Breakspear et al., 2004), the geometric distortions induced by cortical curvature place limitations on the application of wavelet-based methods to contemporary surface-based analyses (Borne et al., 2023). Obtaining geometric eigenmodes from the LBO is a natural extension of orthogonal basis decompositions to curved surfaces and, as shown here, yields surrogate brain maps that preserve SA and provide suitable control of false positives. A related approach, known as Moran spectral randomization (MSR), first weights vertex connectivity of the surface (usually using the inverse of the pairwise distance matrix) and then estimates the graph Laplacian of this matrix. The ensuing eigenfunctions of the generalized eigenvalue problem for the Laplacian are then used to decompose a map on the surface (similar to Eq. 1). Surrogate maps are derived by randomly flipping the (positive or negative) sign of the coefficients (Wagner & Dray, 2015). This process yields $2^{n-1}$ surrogates, where *n* is the total number of eigenfunctions, far fewer than arising from free rotation of geometric eigengroups as in this paper, which is (Λ−1)! Moreover, if the spatial map loads onto a small number of the eigenfunctions, as often happens, flipping the sign of the coefficient yields surrogates that are strongly (anti-)correlated to the original data, producing multimodal null distributions (Burt et al., 2020). As a result, the MSR achieves poorer FPR in similar tests (Markello & Misic, 2021).

Geometric eigenmodes and their associated eigenvalues are obtained by solving the Helmholtz equation on a discrete cortical mesh (see Methods). As such, geometric eigenmodes play a crucial role in generative models of brain activity (Gabay & Robinson, 2017; Roberts et al., 2019; Robinson et al., 2016) and morphology (Cao et al., 2023). In particular, physiologically derived neural field models (Robinson et al., 2005) are separable into their temporal and spatial components under broad assumptions (Gabay & Robinson, 2017; Robinson et al., 2016). The spatial component of a broad class of neural field models satisfies the Helmholtz equation, yielding the geometric modes that we presently employ. These modes thus capture how geometry constrains large-scale neural activity (Pang et al., 2023). The temporal component of neural field models assigns damped oscillations to each eigenmode (higher frequencies are associated with eigenmodes with shorter characteristic wavelengths). Although we use geometric eigenmodes for a specific statistical purpose, this deeper connection to neural field theory (NFT) could assist in linking the statistical inference that eigenstrapping affords to deeper causal inference. By testing the null hypothesis that two maps are not specifically related over and above the expectations of two random maps with matched SA (as this method provides), the exploration of other interpretations is possible. For example, if use of eigenstrapping reveals a significant association between a map of myelin thickness and the spatial topography of power in a particular frequency, the parameters of a neural field model, such as a gain parameter, could be allowed to be spatially dependent. This would incorporate the effect of stronger local feedback. Likewise, excursion from a linear eigenstrapped null could motivate exploration of the nonlinear excitation of NFT, extending prior work from a purely temporal to a spatiotemporal framework (Breakspear et al., 2006; Robinson et al., 2002).

Parametric tests can be used for inference on spatial patterns with relatively simple smoothness, such as those generated by a first order auto-regressive process (Supplementary Information S1). However, functional and cytoarchitectural maps possess complex spatial smoothness, so even the use of multiple spatial kernels (as employed in the BrainSMASH method) fails to capture the full 1/f-like spatial power spectra. Unlike homogeneously smoothed maps, such heavy-tailed processes yield non-trivial (patchy) local roughness (i.e., heterogeneous local smoothness), even if they arise from static models with time-independent coefficients (Roberts et al., 2015). While there is growing interest in the non-stationarity of spatial maps, time-dependent stochastic processes are typically more complex than static models that display heterogeneous (patchy) yet stationary statistics. For instance, variance and covariance can undergo discrete shifts in a hidden Markov model, which is weak-sense stationary (i.e., first and second order statistics are constant in space and/or time) but can resemble non-stationary processes if insufficiently sampled (Liégeois et al., 2017). Likewise, a generalized auto-regressive conditional heteroskedasticity (GARCH) process, as widely used in financial modeling, can yield patchy changes in variance and volatility but is stationary when sufficiently sampled. We explored the performance of eigenstrapping in the presence of a clear discontinuity in SA between two divided halves of the cortical mesh. Eigenstrapping maintained appropriate control of false positives in the presence of moderate-to-large differences in local SA (Fig. 8). While these macroscopic differences likely encompass those present in empirical maps, the employment of eigenstrapping in the presence of heterogeneities could be further developed by using geometric eigenmodes derived from an anisotropic Laplace–Beltrami operators that explicitly accommodates heterogeneities in the underlying diffusion process (Andreux et al., 2015).

Eigenstrapping is a versatile approach, being applicable to both surface- and volume-based analyses. It is also fast for most applications, with 200–500 modes being adequate to randomize common neuroimaging datasets, such as smoothed fMRI maps, while preserving intrinsic spatial structure. We also note that incremental rotation of eigengroups (applying a series of random but small rotations) would allow one to track the gradual randomization of a brain map through a complex morphospace (Avena-Koenigsberger et al., 2015), similar to the approach recently applied to synthetic brain networks (Gollo et al., 2018) and natural images (Puckett et al., 2020). In sum, eigenstrapping offers a flexible methodology for null hypothesis testing and choice of generative models in systems neuroscience, broadening the role of non-parametric analyses of neuroimaging data (Winkler et al., 2016).

## Supplementary Material

Supplementary Material

## Data Availability

All code to reproduce the results of the paper is available at https://github.com/SNG-Newy/eigenstrapping-analysis/. The eigenstrapping method itself is available at https://github.com/SNG-newy/eigenstrapping/ (Koussis & Systems Neuroscience Group, 2024). Complete documentation on the method’s usage is also available from the same location. Raw and preprocessed HCP data can be accessed at https://db.humanconnectome.org/.

## Appendix A: Eigenstrapping Algorithm

The algorithm for rotating eigengroups and creating randomized, SA-preserving surrogate brain maps (the eigenstrapping algorithm; Algorithm 1) is outlined in pseudocode, as follows:

**Table:**

**Algorithm 1.** Eigenstrapping algorithm
Inputs: Brain map data y(x), EITHER surface x OR pre-computed modes Ψ(x) Mask w ← index NaNs in y(x) OR Neumann boundary if x do compute modes Ψ(x[! w]) using FEM method (Reuter et al., 2009) end if Estimate coefficients β from brain map y[! w] using Eq. (7) Derive residuals ε(x[! w]) as in Eq. (7) Index groups Λ← total number of groups (G) (or modes) Initialize new modes array $\Psi^{\prime }=\Psi [!\;\text{w}]$ for i=1:G do generate random rotation matrix ${\text{R}}^{\text{n}\times \text{n}}\leftarrow \text{SO}\left(\text{n}\right)$ $\phi_{i,\mu }=\Psi_{i,\mu }\lambda_{i,\mu }^{\frac{1}{2}}$ $\phi_{i,\mu }^{'}=\phi_{i,\mu }\cdot R^{n\times n}$ $\psi_{i,\mu }^{'}=\phi_{i,\mu }^{'}\lambda_{i,\mu }^{\frac{1}{2}}$ $\psi_{i}^{'}=\{\psi_{i,\mu }^{'}\}$ end for Surrogate map ${\text{y}}^{\prime }[!\;\text{w}]=\beta \Psi^{\prime };{\text{y}}^{\prime }[\text{w}]=\text{NaN}$ (optional) ${\text{y}}^{\prime }[!\;\text{w}]$ amplitude adjusted ← ${\text{y}}^{\prime }[!\;\text{w}][\text{sort}({\text{y}}^{\prime }[!\;\text{w}])]=\text{y}[!\;\text{w}][\text{sort}(\text{y}[!\;\text{w}])]$ (optional) ${\text{y}}^{\prime }[!\;\text{w}]={\text{y}}^{\prime }[!\;\text{w}]+\text{perm}(\in [!\;\text{w}])$

Note that the operator (!) refers to the logical NOT operator. (′) refers to “new,” or “prime,” not the first derivative or the transpose.
