# Supplementary material for: Generation of surrogate brain maps preserving spatial autocorrelation through random rotation of geometric eigenmodes
Source: Imaging Neurosci (Camb). 2025 Jul 16;3:IMAG.a.71. doi: 10.1162/IMAG.a.71 (PMC12330862; doi:10.1162/IMAG.a.71)
Supplement: Supplementary Material [file IMAG.a.71_supp.pdf]

Supplementary Information for

Generation of surrogate brain maps preserving spatial autocorrelation through random rotation of geometric eigenmodes

Nikitas C. Koussis[1,2]*, James C. Pang[3], Richa Phogat[1,4], Jayson Jeganathan[1,4,5], Bryan Paton[1,2,4], Alex Fornito[3], P. A. Robinson[6], Bratislav Misic[7], Michael Breakspear[1,4,5]

Affiliations

[1]Neuromodulation Program, Hunter Medical Research Institute, New Lambton Heights, New South Wales, Australia

[2]Mark Hughes Foundation Centre for Brain Cancer Research, College of Health, Medicine and Wellbeing, University of Newcastle, Callaghan, New South Wales, Australia

[3]School of Psychological Sciences, Turner Institute for Brain and Mental Health, and Monash Biomedical Imaging, Monash University, Clayton, Victoria, Australia

[4]School of Psychological Sciences, College of Engineering, Science, and the Environment, University of Newcastle, Callaghan, New South Wales, Australia

[5]School of Medicine and Public Health, College of Health, Medicine, and Wellbeing, University of Newcastle, Callaghan, New South Wales, Australia

[6]School of Physics, University of Sydney, Camperdown, New South Wales, Australia

[7]Network Neuroscience Lab, Montréal Neurological Institute, McGill University, Montréal, Québec, Canada

*Corresponding author. Email: nikitas.koussis@newcastle.edu.au

Table of Contents

S1. STATISTICAL BACKGROUND 3

S2. PROPERTIES OF GEOMETRIC EIGENMODES 4

S3. TREATMENT OF THE RESIDUALS OF THE EIGENMODE DECOMPOSITION 7

S4. AMPLITUDE ADJUSTMENT 7

S5. HCP DATA 8

S6. GAUSSIAN RANDOM FIELDS 9

S7. MORAN'S *I* 9

S8. CALCULATION OF CORTICO-SUBCORTICAL FUNCTIONAL CONNECTIVITY PATTERNS 10

S9. TREATMENT OF THE MEDIAL WALL 11

S10. GENERATION OF LOCAL TEXTURE PATTERNS 12

S11. SURROGATE COMPUTATION TIME 13

SUPPLEMENTARY REFERENCES 15

SUPPLEMENTARY FIGURES 20

SUPPLEMENTARY TABLES 36

S1. Statistical background

Let two vectors $y(\boldsymbol{x})$ and $z(\boldsymbol{x})$ denote brain maps on a discretized cortical surface X with vertices $\boldsymbol{x}$ with N points. The correlation coefficient ρ_{yz} measures the strength and direction of the linear relationship between these two maps y and z across the surface,

$$\rho_{yz} = \frac{\sum_{i=1}^{N}(y(x_i) - \bar{y}).(z(x_i) - \bar{z})}{\sigma_y \sigma_z}, \qquad (S1)$$

where $\bar{y}$ and $\bar{z}$ are the average values of y and z over $\boldsymbol{x}$; σ_y and σ_z are the corresponding standard deviations and x_i denotes the i-th vertex.

The null hypothesis $\mathcal{H}_0$ that the alignment of the two maps is random implies that the empirical test statistic ρ_{yz} is drawn from a null distribution centered at zero, i.e. $\mathbb{E}(\rho_{yz}) = 0$. If the maps possess no autocorrelation then the variance of the test statistic ρ_{yz} under the null is given by,

$$var(\rho_{yz}) = \frac{1}{N}. \qquad (S2)$$

However, if the maps $y(\boldsymbol{x})$ and $z(\boldsymbol{x})$ possess SA, the effective sample size is reduced, inflating the distribution of the test statistic under the null.

Suppose $y(\boldsymbol{x})$ and $z(\boldsymbol{x})$ are uncorrelated with each other, but autocorrelated first order stochastic processes defined on a one-dimensional domain $\boldsymbol{x} \in \mathbb{R}$. Let them possess autocorrelation functions $r_y(d)$ and $r_z(d)$ computed at discrete lags d. Then the variance of the test statistic ρ_{yz} under the null is approximated by (Afyouni et al., 2019),

$$var(\rho_{yz}) = \frac{1}{N^2}\left[N + 2\sum_{d=1}^{N-1}(N - d)\, r_y(d) r_z(d)\right]. \qquad (S3)$$

For two spatially autocorrelated maps $y(\boldsymbol{x})$ and $z(\boldsymbol{x})$ on a two-dimensional domain $\boldsymbol{x} \in \mathbb{R}^{\mathbf{2}}$, with directionally specific autocorrelation functions $r_y(d_1, d_2)$ and $r_z(d_1, d_2)$ at discrete lags $d_1$and d_2. The formula for the approximate variance of the correlation coefficient under the null is then,

$$var(\rho_{yz}) = \frac{1}{N^2}\left[N + 2\sum_{d_1=1}^{N-1}\sum_{d_2=1}^{N-1}(N - d_1)(N - d_2)\, r_y(d_1, d_2) r_z(d_1, d_2)\right], \qquad (S4)$$

which permits parametric inference for the correlation between two empirical maps, if their properties conform to those of a first order autoregressive stochastic process.

Empirical maps derived from neuroimaging data typically possess complex spatial properties with smoothness that either does not conform to a simple parametric form or is a composite of distinct processes at short versus long spatial distances. Nonetheless, the expected variance of the test statistic under the null can still be estimated from the sample autocorrelations $r_y(d_1, d_2)$ and $r_z(d_1, d_2)$ across all lags d_1 and d_2 using the same equation (S4). Nonparametric, surrogate data methods are useful when estimating this becomes challenging or prohibitive due to long wavelengths and anisotropies in the correlation structure of the data. Surrogate methods use adaptive resampling methods (such as eigenstrapping) to yield surrogate maps $y'(\boldsymbol{x})$ and $z'(\boldsymbol{x})$ on the domain $\boldsymbol{x} \in \mathbb{R}^2$, with sample autocorrelations $r_{y'}$ and $r_{z'}$ that replicate those of the empirical data, i.e. $r_{y'}(d_1, d_2) \approx r_y(d_1, d_2)$ and $r_{z'}(d_1, d_2) \approx r_z(d_1, d_2)$. In this setting the RHS of (S4) is approximately equal for both the real and surrogate data and hence,

$$var(\rho_{y'z'}) \approx var(\rho_{yz}), \qquad (S5)$$

S2. Properties of geometric eigenmodes

Geometric eigenmodes of a surface $\boldsymbol{x}$ are derived from the Laplace-Beltrami operator (LBO); more specifically, the time-invariant solutions to the eigenvalue problem, also known as the Helmholtz equation,

$$\Delta\psi_\eta(\boldsymbol{x}) = -\lambda_\eta \psi_\eta(\boldsymbol{x}), \qquad (S6)$$

where $\Psi = \{\psi_\eta\}_{\eta=0}^{\infty}$ are the eigenmodes, $\{\lambda_\eta\}_{\eta=0}^{\infty}$ are the corresponding eigenvalues, and $\eta = 0,1,2,\ldots$ indices the modes. The geometric eigenmodes $\psi_{\eta=\Lambda\mu} = \{\psi_{0,0}(\boldsymbol{x}), \psi_{1,-1}(\boldsymbol{x}), \psi_{1,0}(\boldsymbol{x}), \ldots, \psi_{\Lambda,\mu}(\boldsymbol{x})\}$ have corresponding eigenvalues $\lambda_{\eta=\Lambda\mu} = \{\lambda_{0,0}, \lambda_{1,-1}, \lambda_{1,0} \ldots, \lambda_{\Lambda,\mu}\}$, where the index of mode η becomes group Λ and number μ. When the Helmholtz equation is applied to study waves, the eigenvalues are typically denoted by $\lambda = k^2$ where k is analogous to the wave number, to which it reduces in the case of a planar cortex (Robinson et al., 2016). These groups increase in size monotonically according to the multiplicity factor $n = 2\Lambda + 1$ and decrease in spatial wavelength with group (Fig. S2). The integer μ in each group Λ ranges from $-\Lambda \leq 0 \leq \Lambda$.

Suppose that the cortical surface X has a continuous boundary ∂X. The LBO has Neumann boundary conditions,

$$\frac{\partial}{\partial \boldsymbol{n}}(\boldsymbol{f}) = 0 \text{ on } \partial X, \qquad (S7)$$

where $\boldsymbol{n}$ denotes the linear subspace that is normal to ∂X and $\boldsymbol{f}$ is a smooth function (such as a cortical map) defined on X.

Perturbation theory can be used to express LBO eigenfunctions ψ_{lm} and eigenvalues λ_{lm} on non-spherical surfaces in terms of first order perturbations of the spherical harmonics (Gabay & Robinson, 2017). For the geometric eigenvalues from Eq. (S6) this corresponds to

$$\lambda_{lm} = \xi_{lm} + \Omega_{lm}\xi'_{lm}, \tag{S8}$$

where ξ_{lm} is the unperturbed (spherical harmonic) eigenvalue and ξ'_{lm} is the first order perturbation with coefficient Ω_{lm}. Hence, this expresses eigenmodes and their eigenvalues as a first order perturbation of spherical harmonics. We use the indexing $\eta = \Lambda\mu$ with group Λ and mode number μ in the geometric (cortical and subcortical) case, and $\eta = lm$ in the spherical case to disambiguate geometric eigenmodes and spherical harmonics.

Crucially, the geometric eigenvalues within groups are perturbed by differing amounts because of the symmetry breaking transformation of the sphere onto the folded cortex (Robinson et al., 2016). That is, the perturbation $\Omega_{lm} \neq \Omega_{ln}$ and thus the eigenvalues $\lambda_{lm} \neq \lambda_{ln}$ when $m \neq n$. Fig. S1 shows the first 16 eigenvalues obtained by solving Eq. (S6) on increasingly folded cortices from a spherical representation (far left; $\rho = 0$) to a fully folded cortex (far right; $\rho = 1$) using FreeSurfer (Fischl et al., 1999), as a function of folding ρ. As ρ increases, the average eigenvalue within groups Λ remains nearly constant (exactly so for the zeroth group $\Lambda = 0$) while the eigenvalues for individual modes demonstrate perturbed energies, splitting but not crossing with modes from adjacent groups.

A spherical mode $\phi_{\Lambda\mu}(\boldsymbol{z})$ on sphere $\boldsymbol{z}$ can be expressed as a weighted sum of spherical harmonics $Y_{lm}(\boldsymbol{z})$,

$$\phi_{\Lambda\mu}(\boldsymbol{z}) = \sum_{l}\sum_{m} C_{\Lambda\mu lm}\, Y_{lm}(\boldsymbol{z}), \tag{S9}$$

where $C_{\Lambda\mu lm}$ are coefficients of this expansion of spherical eigenmodes $\phi_{\Lambda\mu}(\boldsymbol{z})$ in terms of spherical harmonics $Y_{lm}(\boldsymbol{z})$. For low order modes, the terms on the RHS are predominated by harmonics from the same group as the spherical eigenmodes, that is $C_{\Lambda\mu} \gg C_{lm}$ for groups $l \neq \Lambda$ (Robinson et al., 2016). For higher modes, there is greater "leakage" from adjacent groups (Pang et al., 2023b). Note that geometric eigenmodes can be more complex than their corresponding spherical harmonics, such as differing numbers of positive and negative domains within the same group. However, the Courant Nodal Line Theorem limits the complexity of the resulting modes by restricting the number of separate regions that can have positive or negative sign to at most *n* for the *n*th eigenmode (Robinson et al., 2016).

For a specific surface, such as a cortical or hippocampal mesh, the corresponding full set of eigenmode basis functions are uniquely determined by the resolution of the mesh tessellation (i.e. the number N of vertices) and its geometry (curvature) (Seo & Chung, 2011). For a sphere

with N uniformly spaced vertices, there are a maximum of $\Lambda_{max} = \sqrt{N/2} - 1$ groups each with $\mu = 2(\Lambda + 1)$ modes. For a highly resolved mesh, the highest order modes can be vulnerable to aliasing so the number of groups (and hence modes) is generally rounded down to the next integer, i.e. $\Lambda_{max} = floor(\sqrt{N/2} - 1)$. Practically, we typically needed far fewer than the maximum number of modes to retain smoothness following eigenmode rotation (see main text **2.4 Depth of decomposition and treatment of residuals**, p14). We calculated all eigenmodes using the LaPy package implemented in Python (refer to https://github.com/Deep-MI/LaPy).

The composition of these eigengroups and the geometric properties of each ellipsoid are crucial to the eigenstrapping approach. The approximate wavelength on the cortex can be calculated for each cortical eigengroup as (Cao et al., 2023; Pang et al., 2023a)

$$\text{wavelength} \cong \frac{2\pi R_s}{[l(l+1)]^{\frac{1}{2}}}. \qquad (S10)$$

The wavelengths for a sphere of radius $R_s \approx 67$ mm (approximately the radius of the *fsaverage5* population-average template used in this study) are listed in Supplementary Table 1, along with eigengroup membership for the first 1000 modes. The linear relationship of eigengroup membership and the relationship of wavelength to group is given in Fig. S2. Fig. S2 also shows the group size for the first 100 eigengroups, corresponding to the first 10000 modes.

It is also useful to relate the full-width half-maximum (FWHM) of a particular map to the wavelength of eigenmodes on the corresponding surface. We derived a heuristic for Alg. 1 that automates the choice of the initial number of modes, the choice of which is important to replicate the SA of the map.

This heuristic is as follows:

i. Estimate the FWHM of the map (we used Connectome Workbench *wb_command -metric-estimate-fwhm* (Marcus et al., 2011) for this purpose).
ii. Find the number g of whole eigengroups with spatial wavelengths (Eq. S10) longer than 2 × FWHM, satisfying the condition,

$$2 \times \text{FWHM} \leq \frac{2\pi R_s}{[l(l+1)]^{\frac{1}{2}}}, l \in \{1 \dots g\} \qquad (S11)$$

iii. Truncate the number of modes for surrogate map generation (Alg. 1) to g.

We chose condition (ii) above assuming periodicity to the SA. In other words, FWHM is the width of one "crest" in the map, while the wavelength of the modes reflects the length of a "crest" and "trough" together (therefore wavelength = 2xFWHM). The monotonic relationship of FWHM for the first 3000 modes on the *fsaverage5* surface are plotted against their

eigenvalues in Fig. S3. To verify the validity of this approach, we repeated the FPR calculation (i.e., Fig. 3) at different scales of FWHM (1.5, 2.0, 2.5). We find that the control of false positives at 2xFWHM is robust to these modest changes in the heuristic (Fig. S4). Note that following the above procedure provided an improvement to FPR over previous versions of the method (version 2; c.f., Fig. 3D in Koussis et al., 2024).

S3. Treatment of the residuals of the eigenmode decomposition

The eigenmode decomposition,

$$y(\boldsymbol{x}) = \sum_{\Lambda=0}^{G} \sum_{\mu=-\Lambda}^{\Lambda} \left(\beta_{\Lambda\mu}\psi_{\Lambda\mu}(\boldsymbol{x})\right) + \varepsilon(\boldsymbol{x}), \quad \text{(S12)}$$

on a mesh with N vertices on discrete surface $\boldsymbol{x}$ is complete when a total of N-1 eigenmodes are used, whereby the residual term ε limits to zero (to numerical accuracy). However, for a highly resolved cortical mesh (e.g., 32,492 vertex points per hemisphere), a complete representation of n=N-1 modes per hemisphere carries a substantial computational burden. As power diminishes at higher spatial frequencies, incomplete decompositions typically yield unstructured residual error for $n \ll N$. Residuals are calculated by subtracting the reconstructed data from the original data. Despite their relatively small coefficients, they contain substantial variance that needs to be included in the surrogate data (Fig. S4C). These residuals can be added back into the surrogates directly, resulting in near-exact matching of the variogram even when resampling a small number of modes relative to the total number on the *fs-LR-32k* surface (n ~ 200, corresponding to <1% of all modes; Fig. SA). However, this re-addition of the original residual leads to sub-optimal behavior of the resulting surrogate maps, such that they are correlated with the original data and each other (Fig. S4B), due to the perfect matching of these small terms between the original and all the surrogate maps.

To retain the variance without yielding pairwise correlated surrogates, the residuals can be *permuted* before being added to the surrogates (Fig. S). However, the combined effects of adding the permuted residuals and undertaking amplitude adjustment (see below) only preserves SA if the number of modes is sufficiently high (Fig. SC).

S4. Amplitude adjustment

Amplitude adjustment (AA) is commonly used in Fourier decomposition and wavelet-based surrogate techniques for time-series analysis when the original data does not possess a normal amplitude distribution (Breakspear et al., 2003; Lancaster et al., 2018; Schreiber & Schmitz, 1996, 2000). This step is required to preserve the original distribution of the data, because, due to the Central Limit Theorem, resampling methods yield surrogate data with a Gaussian amplitude distribution, hence causing a mismatch with empirical data sets that have non Gaussian amplitude distribution (Breakspear et al., 2004; Patel et al., 2006; Schreiber &

Schmitz, 1996, 2000) (Fig. 1, panel C). AA is performed by rank-sorting the surrogate data and the empirical data, then replacing the highest-ranked values of the surrogate data with the highest-ranked values of the empirical data, then the next highest, and so on, until all the data have been replaced, ensuring that the surrogate map has exactly the same amplitude distribution as the original data (i.e., the same histogram).

As evident in Fig. S, the AA has a small but non-negligible impact on the variogram. This subtle whitening effect (increasing the variance at small separations) is a known effect when AA is applied to Fourier or wavelet-based resampling techniques and arises due to the re-sorting and re-insertion of the original data samples (Breakspear et al., 2004; Lancaster et al., 2018; Schreiber & Schmitz, 1996, 2000). In the present setting, this effect means that the final number of modes, the application of AA and the need to permute the residuals before re-insertion are somewhat co-dependent. These dependencies can be mitigated when using a sufficient number of modes to generate surrogate data. Indeed, if AA is performed after a sufficient number of modes is rotated, the variance of the surrogate map is very nearly equal to the variance of the original map and the permutation of the residual term may not be necessary. While performing AA or not seems to nominally affect the FPR of the method, performing residual permutation when SA is present is not advised, as it can inflate false positives (Fig. S7).

In our testing we recommend a conservative estimate of minimum 200 modes for standard fMRI datasets with voxel resolutions ≥ 2 mm and smoothing kernels of 4 mm full-width half-maximum (FWHM) and greater. We also offer a tool to automate the selection of the number of modes based on estimation of FWHM of the data, in the function *eigenstrapping.geometry.find_fwhm* (see Supplementary Information-S2).

S5. HCP data

All preprocessed fMRI data was accessed from the Human Connectome Project (Van Essen et al., 2013). No further preprocessing steps were applied, and data was analyzed from 255 unrelated healthy individuals (aged 22-35 years, 132 females and 123 males). This is the largest cohort of the HCP excluding twins or siblings that had completed all tasks and resting-state acquisitions. All procedures entailed in this study were carried out in accordance with local ethics guidelines and with approval from the local ethics committee (University of Newcastle HREC ref: H-2020-0443). Image acquisition parameters, task protocols, and preprocessing pipelines are thoroughly detailed in refs. (Glasser et al., 2013; Van Essen et al., 2013).

Seven task domains detailed in Supplementary Table 2 and task-free resting-state fMRI were analyzed in our study, already preprocessed by HCP. Volumetric contrast activation maps for tasks were resampled to *fs-LR-32k* CIFTI space using Connectome Workbench tools. Volumetric timeseries for resting-state data were used for the cortico-subcortical connectivity gradients. Further information on the construction of these gradients can be found in Supplementary Information-S8.

S6. Gaussian random fields

GRF pairs were generated by adapting Python code from https://github.com/markello_spatialnulls/parspin, using the method described in (Markello & Misic, 2021). First, two uniformly spaced three-dimensional grids with tiling corresponding to the dimensions of the MNI152 2mm standard volume (91x107x91) were generated from a Gaussian distribution. These were then Fourier transformed, colored by parameter α, and inverse Fourier transformed back to MNI152 space. Each pair was then normalized to have zero mean and unit variance. The normalized pairs were then projected to the *fsaverage5* surface with 10,242 vertices using *neuromaps transforms.mni152_to_fsaverage* (Markello et al., 2022).

The unsmoothed histogram of correlations between random pairs of GRFs for each α from 0.0 to 4.0 are provided in Fig. S. This shows that while the correlations are zero-centered, the tails of the distributions widen as α increases.

S7. Moran's *I*

Moran's *I* is a measure of the autocorrelation of spatial data, commonly utilized in geostatistics and texture analysis (Anselin, 1995; Moran, 1950). Unlike the variogram, Moran's *I* is not distance dependent, but rather a composite of SA across all pairwise distances.

Moran's I of a function $f_i = f(\boldsymbol{x_i})$ on a discrete surface $\boldsymbol{x_i}$ with distance weights $w_{ij} = 1/|\boldsymbol{x_i} - \boldsymbol{x_j}|$ is defined as,

$$I = \frac{N}{W} \frac{\sum_{i=1}^{N} \sum_{j=1}^{N} w_{ij} (f_i - \bar{f})(f_j - \bar{f})}{\sum_{i=1}^{N} (f_i - \bar{f})^2}, \tag{S13}$$

where N is the total number of vertices on $\boldsymbol{x}$, $\bar{f}$ is the mean of f, w_{ij} are the elements of distance weights where $w_{i=j,j=i} = 0$ and $W = \sum_{i=1}^{N} \sum_{j=1}^{N} w_{ij}$ (Anselin, 1995).

We benchmarked the SA-replicating property of eigenstrapping (blue) against BrainSMASH (green) and Spin Test (yellow) in Fig. S. Evaluating the performance of each method was achieved by calculating the change in the Moran's I ($\Delta I = empirical\ I - null\ I$) statistic between 1000 simulated maps (GRFs) and 1000 surrogates for each value of α across a range from 0.0 – 3.0 (Fig. S10). A pairwise distance matrix with shape (10242x10242) was derived for the surface that all GRFs were mapped to (*fsaverage5*; see Supplementary Information-S5) and inverted to derive distance weights (the diagonal was set to zero). This weight matrix was then used to calculate Moran's *I*.

Low values of α (0.0 and 1.0) yielded spatial maps with little SA and hence the Moran's I surrounded zero (cyan; Fig. S10A) or was slightly positive (Fig. S10B). All three surrogate methods performed reasonably in this range, with the change in Moran's I (ΔI) centered at zero. Higher values of α generated smoother spatial maps with a corresponding increase in Moran's I (Fig. S10C-E). Notably, surrogates derived using the BrainSMASH method are considerably whiter than the original maps (Moran's I lower than the source map, green). Both eigenstrapping and the spin test preserve the smoothness appropriately – the difference in Moran's I is centered around zero (blue and yellow). Raw Moran's I values are plotted accordingly in Fig. S11 to show the notable decrease in SA of BrainSMASH surrogates.

S8. Calculation of cortico-subcortical functional connectivity patterns

Connection topographical patterns (commonly known as "gradients") of resting-state activity capture the similarity of the functional connectivity patterns of neighboring voxels and possess complex SA(Haak et al., 2018). Calculation of these cortico-subcortical functional connectivity gradients followed a previously published methodology (Haak et al., 2018). Cortico-subcortical functional connectivity gradients were derived by correlating the timeseries of BOLD in the subcortical mask (either thalamus, hippocampus, or striatum) with the principal components (PCs) in whole brain gray matter (GM). Subcortical masks were derived by binarizing the Harvard-Oxford subcortical atlas at 25% probability for each region. PCs were calculated by singular-value decomposition (SVD) of the timeseries, of shape ($T \times T - 1$, where T is the number of volumes). PCs were then correlated with the columns of subcortical activity over time (of shape ($N \times T$) where N is the number of voxels in subcortical GM). The correlation of cortical SVD components and subcortical voxels results in a correlation matrix $\boldsymbol{C}$ of shape ($N \times T - 1$). This correlation matrix was then Fisher's Z-transformed, yielding a positive normal distribution of values between 0 and 1. We characterized the functional connectivity similarity of every subcortical voxel to every other subcortical voxel by deriving the η^2 coefficient row-wise of $\boldsymbol{C}$, resulting in a symmetric similarity matrix $\boldsymbol{S}$,

$$S_{\alpha,\beta} = 1 - \frac{\sum_{j=1}^{p}\left[\left(C_{\alpha,j} - \mu_j\right)^2 - \left(C_{\beta,j} - \mu_j\right)^2\right]}{\sum_{j=1}^{p}\left[\left(C_{\alpha,j} - \bar{\mu}_j\right)^2 - \left(C_{\beta,j} - \bar{\mu}_j\right)^2\right]}, \tag{S14}$$

where the matrix $\boldsymbol{S}$ has rows and columns (α, β) of shape $(V \times V)$. V is the number of voxels in the subcortex, j is the column of the correlation matrix $\boldsymbol{C}$, p corresponds to the total number of columns (the SVD-components), $\mu_j = \frac{C_{\alpha,j} - C_{\beta,j}}{2}$, and $\bar{\mu}$ is the mean of all μ across all p SVD-components.

To derive connectopic gradients, the similarity matrix $\boldsymbol{S}$ must first be made into sparse matrix $\boldsymbol{W}$ to calculate the graph Laplacian. $\boldsymbol{S}$ was rendered sparse according to the following rule for each element of $\boldsymbol{W}$,

$$W_{i,j} = \begin{cases} S_{i,j} & \text{if} \left|\left|S_i - S_j\right|\right|^2 < \varepsilon \\ 0 & \text{if} \left|\left|S_i - S_j\right|\right|^2 \geq \varepsilon \end{cases}, \tag{S15}$$

where ε is the minimum value required for the graph to remain connected. The graph Laplacian $\boldsymbol{L}$ is then calculated by $\boldsymbol{L} = \boldsymbol{D} - \boldsymbol{W}$, where $\boldsymbol{D}$ is equal to the trace of $\boldsymbol{W}$. The eigenvalues and eigenvectors were derived from the generalized eigenvalue problem,

$$\boldsymbol{Lu} = -\zeta \boldsymbol{Du}, \tag{S16}$$

where Δ is the Laplacian operator, ζ are the eigenvalues, and $\boldsymbol{u}$ are the connectopic eigenfunctions corresponding to the eigenvalues. These eigenfunctions are maps wherein voxels with similar values have similar connectivity patterns, and voxels with different values have different connectivity patterns (Haak et al., 2018; Marquand et al., 2017; Tian et al., 2020). The SA of these patterns reflects the typically gradual change across cortex and subcortex of these functional connectivity patterns, with abrupt changes limited to functional boundaries (Tian et al., 2020).

Eigenmodes for resampling were derived from tetrahedral mesh representations of the subcortical structures and projected to the 3D MNI152 space used in this analysis. Unlike the cortex, which can be modeled as a 2D sheet, subcortical volumes are solid 3D objects. Eigenmodes decompositions on 2D surfaces in 3D can be extendable to 3D structures (Wachinger et al., 2015). Volumetric modes were first derived by creating meshes of the volumes (25% probability maps for the Harvard-Oxford atlas for each subcortical structure) by using the marching cubes algorithm *mri_mc* (Henschel et al., 2020) to tessellate the volume, and *Gmsh* (Geuzaine & Remacle, 2009) to derive a tetrahedral mesh from this tessellation.

Recently the hippocampus has also been modeled as a 2D sheet with several layers (DeKraker et al., 2022, 2024). At the time of our analyses, the method for resampling from the volume to the 2D space in (DeKraker et al., 2024) did not exist yet, and so we performed all analyses in Fig. 6 in 3D.

S9. Treatment of the medial wall

Spatial permutation tests such as the Spin Test proceed by rotation of the cortical surface on the sphere, resulting in a substantial number of "medial wall" vertices labeled as non-data (or *unknown*, as FreeSurfer labels it) being re-located to the cortex, with the same number of cortical vertices being rotated onto the medial wall.

There are three general solutions to this problem: (1) Masking out any vertices that are lost (*i.e.*, old medial wall plus new medial wall), that is removing them from any proceeding analyses. This can remove up to 20-30% of the total number of samples on the surface. (2)

Setting all values that lie within the medial wall to zero. (3) Interpolating values across medial wall vertices that are rotated into the new cortical surface. All three solutions bias further analyses to varying degrees, though the most widely used option is (1), which is what this study used to compare in the main paper. The presence of missing data is indicated by the black marker in Fig. 5A.

Eigenstrapping surmounts this issue by setting a Neumann boundary (Eq. S7) around the medial wall and limiting the LBO operator (and thus the modes) to the ensuing closed surface across the cortex. This ensures that rotations are performed in the modal space $\boldsymbol{z}$, not vertex space.

S10. Generation of local texture patterns

A 1024×1024 pixel grayscale image of a face (derived from (Karras et al., 2020); Fig. 7A, *Natural scene*) was projected to the *fsaverage5* cortical surface (10,242 vertices) using a simple (inverse)-stereographic projection (Fig. 7A, *Projection to cortex*). The edges of the image joined at the central sulcus. Eigenstrapping (blue) with 5000 modes solved on the cortical surface (including the medial wall) was used to produce 1000 surrogates. The Spin Test (yellow) was used to rotate the data on the cortical surface 1000 times, producing 1000 rotated surrogates. The medial wall was included in this analysis in order to preserve the original luminance histogram and distortion induced by the projection. Cortical data (original image and surrogates) were then projected to the grid (*Back-projection*) by inverting the initial projection, interpolating with a bilinear spline across non-data pixels induced by projection to the grid.

We employed a local ternary pattern (LTP) as a test statistic for the presence of natural scene statistics. Central pixels c_i with index i were discretized into three values $LTP_i = (-1,0,1)$ by a threshold k on 8-neighbor pixels p_i

$$LTP_i = \begin{cases} 1, & \text{if } p_i > c_i + k \\ 0, & \text{if } p_i > c_i - k \, . \\ -1 & \text{if } p_i < c_i - k \end{cases} \qquad (S17)$$

We used a threshold of k = 5 which is commonly used for face detection algorithms (Gupta et al., 2010; Tan & Triggs, 2007). Each pixel then had a value ranging from -8 to 8, which were then thresholded to positive non-zero values, indicating a common feature to a particular 9-pixel neighborhood. Each of these values were summed, producing a single LTP ($\sum LTP$) for each image. If the texture is disrupted, then the LTP from surrogate maps will be different to the original image. The proportion change in LTP (ΔLTP) was quantified by the equation,

$$\Delta LTP = \frac{empirical \, \sum LTP - surrogate \sum LTP}{empirical \, \sum LTP}. \qquad (S18)$$

To test for a difference in the impact of the two surrogate methods on complex patterns, a Student's T-test was performed on ΔLTP of each method and two-tailed p-values derived.

S11. Surrogate computation time

The time taken for one CPU/thread to compute 1000 surrogates on several different levels of surface density (from volumetric and surface-based analyses), with and without pre-computed eigenmodes is provided in Fig. S14 (specifications for the computational device used for testing are detailed in Supplementary Table S3).

For subcortical maps (*volumetric*), precomputing and caching eigenmodes makes no difference in computation time ("no caching" and "with caching"). In the two cortical surfaces used in this study (*fs-LR-32k* and *fsaverage5*, with 32,492 and 10,242 vertices respectively), the computation of eigenmodes slightly increases the computation time, particularly on the denser *fs-LR-32k* surface, adding on average 50 seconds to the runtime. When the modes have been pre-computed, the two surfaces take on average 250 seconds and 1200 seconds to compute 1000 surrogates. Deriving more modes requires more computation time as the complexity of the mode calculation increases linearly with the surface size (Lehoucq et al., 1998).

The most computationally intensive component of the algorithm (Alg. 1) is a *for* loop that generates $n \times n$ random rotation matrices and performs dot product operations on these. Here, the complexity is no greater than $\mathcal{O}(n^2)$, where n is the number of modes in the largest group (the size of the largest group in the first 10,000 modes is 201; Fig. S). This simplifies even further to $\mathcal{O}(N)$ (where N is the number of vertices) when the rotated modes have been pre-computed.

To overcome the challenge of computation of modes for standard surfaces, a selection of pre-computed modes is integrated into the open-release Python package. The precomputation of modes for standard surfaces should be suitable for the end-user in the majority of cases, as group-level neuroimaging analyses (where null hypothesis testing would generally occur) require registration to a standard space.

One obvious improvement to the computation time is to provide precomputed rotated modes. However, the main limitation of this is the prohibitively large size of these matrices. For instance, a 1000x64984x1000 matrix of 1000 rotated modes Ψ', suitable for left and right hemispheres in *fs-LR-32k* space, stored with double precision, is approximately 200 GB. This makes pre-rotated modes prohibitive to store and make available for many users. However, it is possible to derive precomputed *permutation matrices* for modal rotations. These permutation matrices are derived through nearest-neighbor indexing of rotated modes that reindexes the values of the initial set of eigenmodes. Given that the modes undergo a smooth and reversible transformation in the rotation, this transformation should be equivalent to the rotation steps, only significantly quicker.

The dot product of the modes and permutation matrices results in a relocation of their values on the cortex, as though they had been rotated, without performing the computationally intensive rotation step. This leads to a speed-up of 5-50x depending on the density of the surface and the number of modes (Fig. S15; compare Fig. S14). The procedure for deriving these permutation matrices is as follows:

(i) Calculate rotated modes Ψ' as in Alg. (1) as normal,

(ii) For each modal group column vectors $\Psi'_{\Lambda j}$ with index j, use a nearest neighbor-tree (we used Scikit-Learn's *sklearn.neighbors.BallTree* (Pedregosa et al., 2011)) to find the nearest column vector k in the original modal group Ψ_{Λ}.

(iii) Return the indexing vector that maps $k \rightarrow j$.

(iv) Derive permutation matrix M_i, where each column of M_i is equal to the indexing vectors derived from steps (ii.-iii.), returning an $(N \times G)$ matrix of integers, where N is the number of vertices on the surface and G is the number of groups.

(v) Compute (i.-iv.) for as many permutations P as needed (e.g., 1000), producing $M_P = \{M_1 \ldots M_i\}$.

M_P is an integer array much reduced in size from the full rotated set (the equivalent 1000x64984x31 group permutation matrix M, used to derive the rotated modes as in the example above, is approximately 4 GB). We have integrated a selection of precomputed permutation matrices into the Python package. The user can download these permutation matrices and compute Ψ'_P by reindexing Ψ, per group per permutation, based on each group indexing vector (the last dimension in M_P). This process takes between 20 seconds and 5 minutes for standard surfaces depending on the number of modes (Fig. S15).

As the permutation matrices are derived by nearest neighbor interpolation, there may be some loss of precision. We leave the decision to implement either method up to the user: if precision over speed is important, then use the slower full method. If not, then use the faster permutation method.

Supplementary References

Afyouni, S., Smith, S. M., & Nichols, T. E. (2019). Effective degrees of freedom of the Pearson's correlation coefficient under autocorrelation. *NeuroImage*, *199*, 609–625. https://doi.org/10.1016/j.neuroimage.2019.05.011

Anselin, L. (1995). Local Indicators of Spatial Association—LISA. *Geographical Analysis*, *27*(2), 93–115. https://doi.org/10.1111/j.1538-4632.1995.tb00338.x

Breakspear, M., Brammer, M. J., Bullmore, E. T., Das, P., & Williams, L. M. (2004). Spatiotemporal wavelet resampling for functional neuroimaging data. *Human Brain Mapping*, *23*(1), 1–25. https://doi.org/10.1002/hbm.20045

Breakspear, M., Brammer, M., & Robinson, P. A. (2003). Construction of multivariate surrogate sets from nonlinear data using the wavelet transform. *Physica D: Nonlinear Phenomena*, *182*(1), 1–22. https://doi.org/10.1016/S0167-2789(03)00136-2

Cao, T., Pang, J. C., Segal, A., Chen, Y.-C., Aquino, K. M., Breakspear, M., & Fornito, A. (2023). *Mode-based morphometry: A multiscale approach to mapping human neuroanatomy* (p. 2023.02.26.529328). bioRxiv. https://doi.org/10.1101/2023.02.26.529328

DeKraker, J., Cabalo, D. G., Royer, J., Khan, A. R., Karat, B., Benkarim, O., Rodriguez-Cruces, R., Frauscher, B., Pana, R., Hansen, J. Y., Misic, B., Valk, S. L., Lau, J. C., Kirschner, M., Bernasconi, A., Bernasconi, N., Muenzing, S., Axer, M., Amunts, K., … Bernhardt, B. C. (2024). *HippoMaps: Multiscale cartography of human hippocampal organization* (p. 2024.02.23.581734). bioRxiv. https://doi.org/10.1101/2024.02.23.581734

DeKraker, J., Haast, R. A., Yousif, M. D., Karat, B., Lau, J. C., Köhler, S., & Khan, A. R. (2022). Automated hippocampal unfolding for morphometry and subfield

segmentation with HippUnfold. *eLife*, *11*, e77945. https://doi.org/10.7554/eLife.77945

Fischl, B., Sereno, M. I., Tootell, R. B., & Dale, A. M. (1999). High-resolution intersubject averaging and a coordinate system for the cortical surface. *Human Brain Mapping*, *8*(4), 272–284. https://doi.org/10.1002/(sici)1097-0193(1999)8:4<272::aid-hbm10>3.0.co;2-4

Gabay, N. C., & Robinson, P. A. (2017). Cortical geometry as a determinant of brain activity eigenmodes: Neural field analysis. *Physical Review E*, *96*(3), 032413. https://doi.org/10.1103/PhysRevE.96.032413

Geuzaine, C., & Remacle, J.-F. (2009). Gmsh: A 3-D finite element mesh generator with built-in pre- and post-processing facilities. *International Journal for Numerical Methods in Engineering*, *79*(11), 1309–1331. https://doi.org/10.1002/nme.2579

Glasser, M. F., Sotiropoulos, S. N., Wilson, J. A., Coalson, T. S., Fischl, B., Andersson, J. L., Xu, J., Jbabdi, S., Webster, M., Polimeni, J. R., Van Essen, D. C., Jenkinson, M., & WU-Minn HCP Consortium. (2013). The minimal preprocessing pipelines for the Human Connectome Project. *NeuroImage*, *80*, 105–124. https://doi.org/10.1016/j.neuroimage.2013.04.127

Gupta, R., Mittal, A., & Patil, H. (2010). Robust order-based methods for feature description. In *2010 IEEE Conference on Computer Vision and Pattern Recognition (CVPR)* (pp. 334–341). IEEE Computer Society. https://doi.ieeecomputersociety.org/10.1109/CVPR.2010.5540195

Haak, K. V., Marquand, A. F., & Beckmann, C. F. (2018). Connectopic mapping with resting-state fMRI. *NeuroImage*, *170*, 83–94. https://doi.org/10.1016/j.neuroimage.2017.06.075

Henschel, L., Conjeti, S., Estrada, S., Diers, K., Fischl, B., & Reuter, M. (2020). FastSurfer—A fast and accurate deep learning based neuroimaging pipeline. *NeuroImage*, *219*, 117012. https://doi.org/10.1016/j.neuroimage.2020.117012

Karras, T., Laine, S., Aittala, M., Hellsten, J., Lehtinen, J., & Aila, T. (2020). *Analyzing and Improving the Image Quality of StyleGAN* (No. arXiv:1912.04958). arXiv. https://doi.org/10.48550/arXiv.1912.04958

Lancaster, G., Iatsenko, D., Pidde, A., Ticcinelli, V., & Stefanovska, A. (2018). Surrogate data for hypothesis testing of physical systems. *Physics Reports*, *748*, 1–60. https://doi.org/10.1016/j.physrep.2018.06.001

Leech, R., Smallwood, J. S., Moran, R., Jones, E. J. H., Vowles, N., Leech, D., Viegas, E. M., Turkheimer, F. E., Alberti, F., Margulies, D., Jefferies, E., Bernhardt, B., & Váša, F. (2024). *The impact of heterogeneous spatial autocorrelation on comparisons of brain maps* (p. 2024.06.14.598987). bioRxiv. https://doi.org/10.1101/2024.06.14.598987

Lehoucq, R. B., Sorensen, D. C., & Yang, C. (1998). *ARPACK Users' Guide: Solution of Large-scale Eigenvalue Problems with Implicitly Restarted Arnoldi Methods*. SIAM.

Marcus, D., Harwell, J., Olsen, T., Hodge, M., Glasser, M., Prior, F., Jenkinson, M., Laumann, T., Curtiss, S., & Van Essen, D. (2011). Informatics and Data Mining Tools and Strategies for the Human Connectome Project. *Frontiers in Neuroinformatics*, *5*. https://doi.org/10.3389/fninf.2011.00004

Markello, R. D., Hansen, J. Y., Liu, Z.-Q., Bazinet, V., Shafiei, G., Suárez, L. E., Blostein, N., Seidlitz, J., Baillet, S., Satterthwaite, T. D., Chakravarty, M. M., Raznahan, A., & Misic, B. (2022). neuromaps: Structural and functional interpretation of brain maps. *Nature Methods*, *19*(11), 1472–1479. https://doi.org/10.1038/s41592-022-01625-w

Markello, R. D., & Misic, B. (2021). Comparing spatial null models for brain maps. *NeuroImage*, *236*, 118052. https://doi.org/10.1016/j.neuroimage.2021.118052

Marquand, A. F., Haak, K. V., & Beckmann, C. F. (2017). Functional corticostriatal connection topographies predict goal-directed behaviour in humans. *Nature Human Behaviour*, *1*(8), Article 8. https://doi.org/10.1038/s41562-017-0146

Moran, P. A. P. (1950). Notes on Continuous Stochastic Phenomena. *Biometrika*, *37*(1/2), 17–23. https://doi.org/10.2307/2332142

Pang, J. C., Aquino, K. M., Oldehinkel, M., Robinson, P. A., Fulcher, B. D., Breakspear, M., & Fornito, A. (2023a). Geometric constraints on human brain function. *Nature*, *618*(7965), Article 7965. https://doi.org/10.1038/s41586-023-06098-1

Pang, J. C., Aquino, K. M., Oldehinkel, M., Robinson, P. A., Fulcher, B. D., Breakspear, M., & Fornito, A. (2023b). *Reply to: Commentary on Pang et al. (2023) Nature* (p. 2023.10.06.560797). bioRxiv. https://doi.org/10.1101/2023.10.06.560797

Patel, R. S., Van De Ville, D., & DuBois Bowman, F. (2006). Determining significant connectivity by 4D spatiotemporal wavelet packet resampling of functional neuroimaging data. *NeuroImage*, *31*(3), 1142–1155. https://doi.org/10.1016/j.neuroimage.2006.01.012

Pedregosa, F., Varoquaux, G., Gramfort, A., Michel, V., Thirion, B., Grisel, O., Blondel, M., Prettenhofer, P., Weiss, R., Dubourg, V., Vanderplas, J., Passos, A., Cournapeau, D., Brucher, M., Perrot, M., & Duchesnay, É. (2011). Scikit-learn: Machine Learning in Python. *Journal of Machine Learning Research*, *12*(85), 2825–2830.

Robinson, P. A., Zhao, X., Aquino, K. M., Griffiths, J. D., Sarkar, S., & Mehta-Pandejee, G. (2016). Eigenmodes of brain activity: Neural field theory predictions and comparison with experiment. *NeuroImage*, *142*, 79–98. https://doi.org/10.1016/j.neuroimage.2016.04.050

Schreiber, T., & Schmitz, A. (1996). Improved Surrogate Data for Nonlinearity Tests. *Physical Review Letters*, *77*(4), 635–638. https://doi.org/10.1103/PhysRevLett.77.635

Schreiber, T., & Schmitz, A. (2000). Surrogate time series. *Physica D: Nonlinear Phenomena*, *142*(3), 346–382. https://doi.org/10.1016/S0167-2789(00)00043-9

Seo, S., & Chung, M. K. (2011). Laplace-Beltrami eigenfunction expansion of cortical manifolds. *2011 IEEE International Symposium on Biomedical Imaging: From Nano to Macro*, 372–375. https://doi.org/10.1109/ISBI.2011.5872426

Tan, X., & Triggs, B. (2007). Enhanced Local Texture Feature Sets for Face Recognition Under Difficult Lighting Conditions. In S. K. Zhou, W. Zhao, X. Tang, & S. Gong (Eds.), *Analysis and Modeling of Faces and Gestures* (pp. 168–182). Springer. https://doi.org/10.1007/978-3-540-75690-3_13

Tian, Y., Margulies, D. S., Breakspear, M., & Zalesky, A. (2020). Topographic organization of the human subcortex unveiled with functional connectivity gradients. *Nature Neuroscience*, *23*(11), Article 11. https://doi.org/10.1038/s41593-020-00711-6

Van Essen, D. C., Smith, S. M., Barch, D. M., Behrens, T. E. J., Yacoub, E., Ugurbil, K., & WU-Minn HCP Consortium. (2013). The WU-Minn Human Connectome Project: An overview. *NeuroImage*, *80*, 62–79. https://doi.org/10.1016/j.neuroimage.2013.05.041

Wachinger, C., Golland, P., Kremen, W., Fischl, B., Reuter, M., & Alzheimer's Disease Neuroimaging Initiative. (2015). BrainPrint: A discriminative characterization of brain morphology. *NeuroImage*, *109*, 232–248. https://doi.org/10.1016/j.neuroimage.2015.01.032

Supplementary Figures

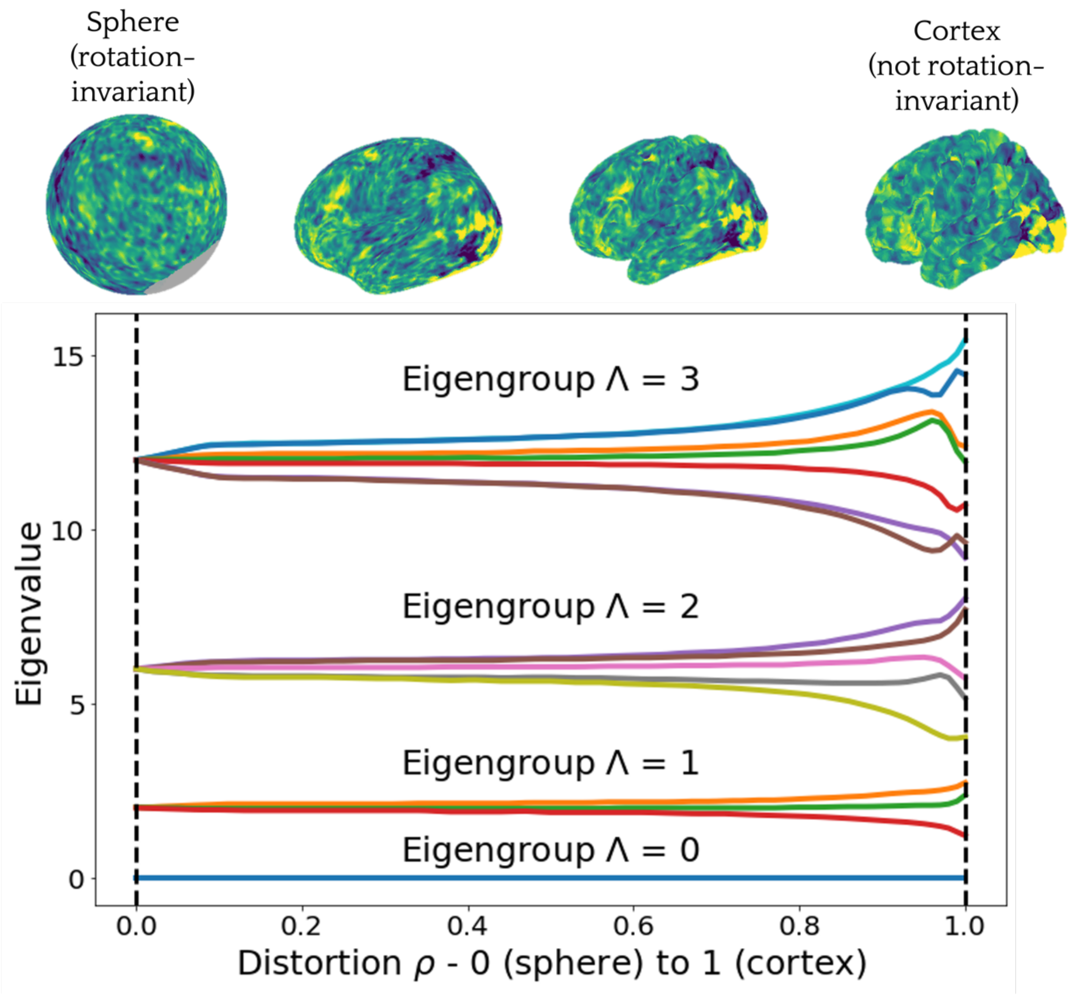

Fig. S1. Eigenvalue spectrum of the first 4 eigengroups on the left hemisphere, corresponding to the first 16 eigenvalues. The spectrum is evaluated at 100 points as the cortical surface (ρ = 1.0) is gradually inflated to the sphere (ρ = 0.0). The degree of distortion is proportional to the folding of the cortex. Note that the inflation of the surface was fixed so that surface area was kept consistent across surfaces. Eigenvalue figure adapted with permission from (Pang et al., 2023b; Robinson et al., 2016).

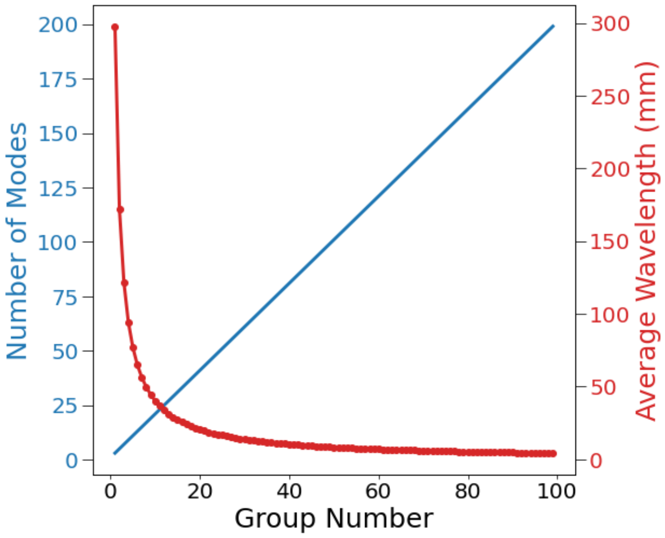

Fig. S2. Number of eigenmodes per eigengroup (blue) and wavelength in mm of each eigengroup (red) up to the first 100 eigengroups (corresponding to the first 10000 modes).

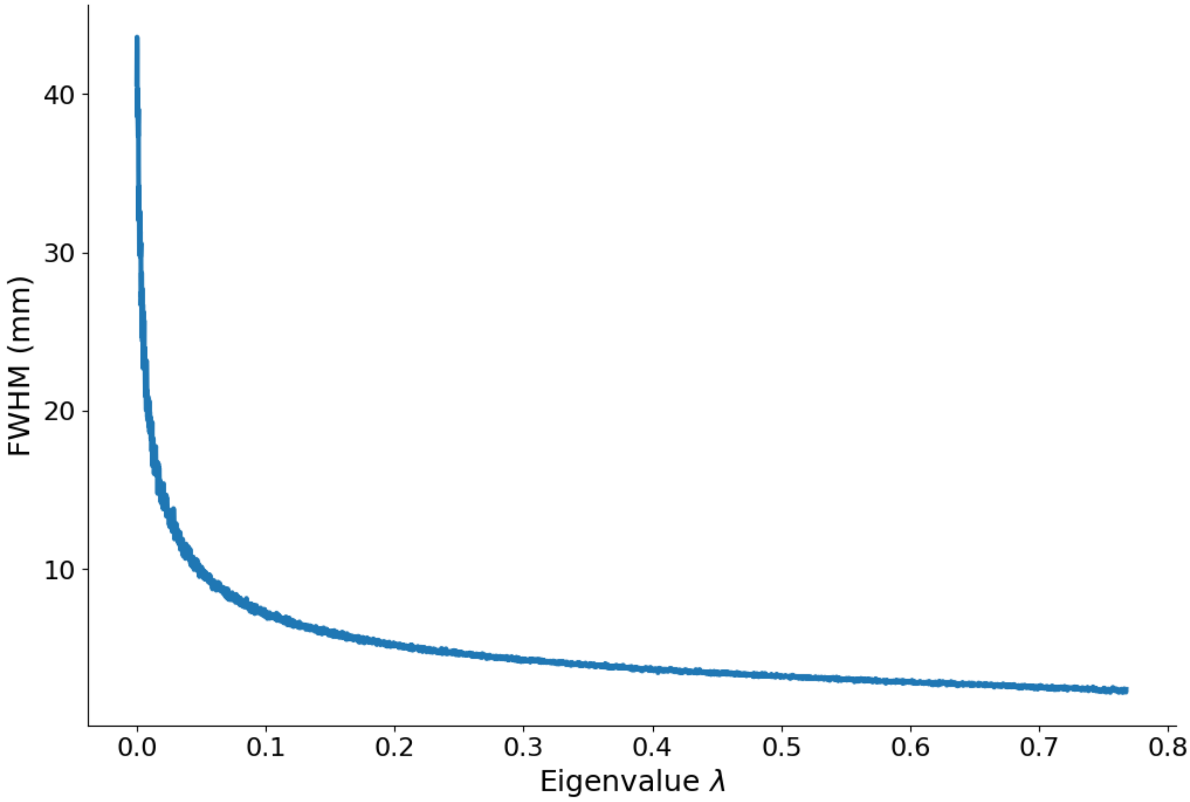

Fig. S3. Monotonic relationship of modal full-width half-maxima (FWHM) and eigenvalue. The first 3000 modes on the *fsaverage5* surface with 10k vertices were calculated. As the eigenvalue increases, the spatial and temporal frequency of the modes increases.

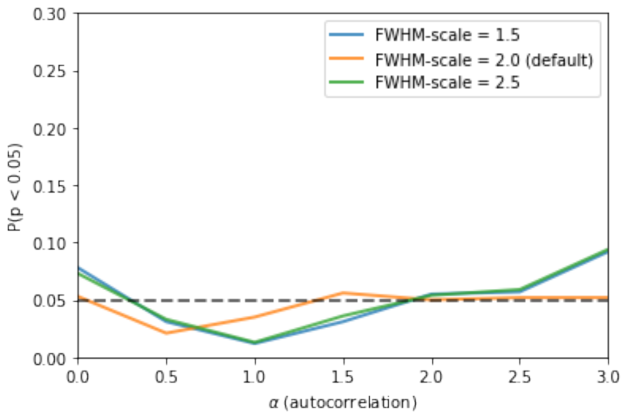

Fig. S4. The effect of FWHM scales on eigenstrapping false positives. FWHM-scale = 1.5 shown in blue; FWHM-scale = 2.0 shown in orange; FWHM-scale = 2.5 shown in green.

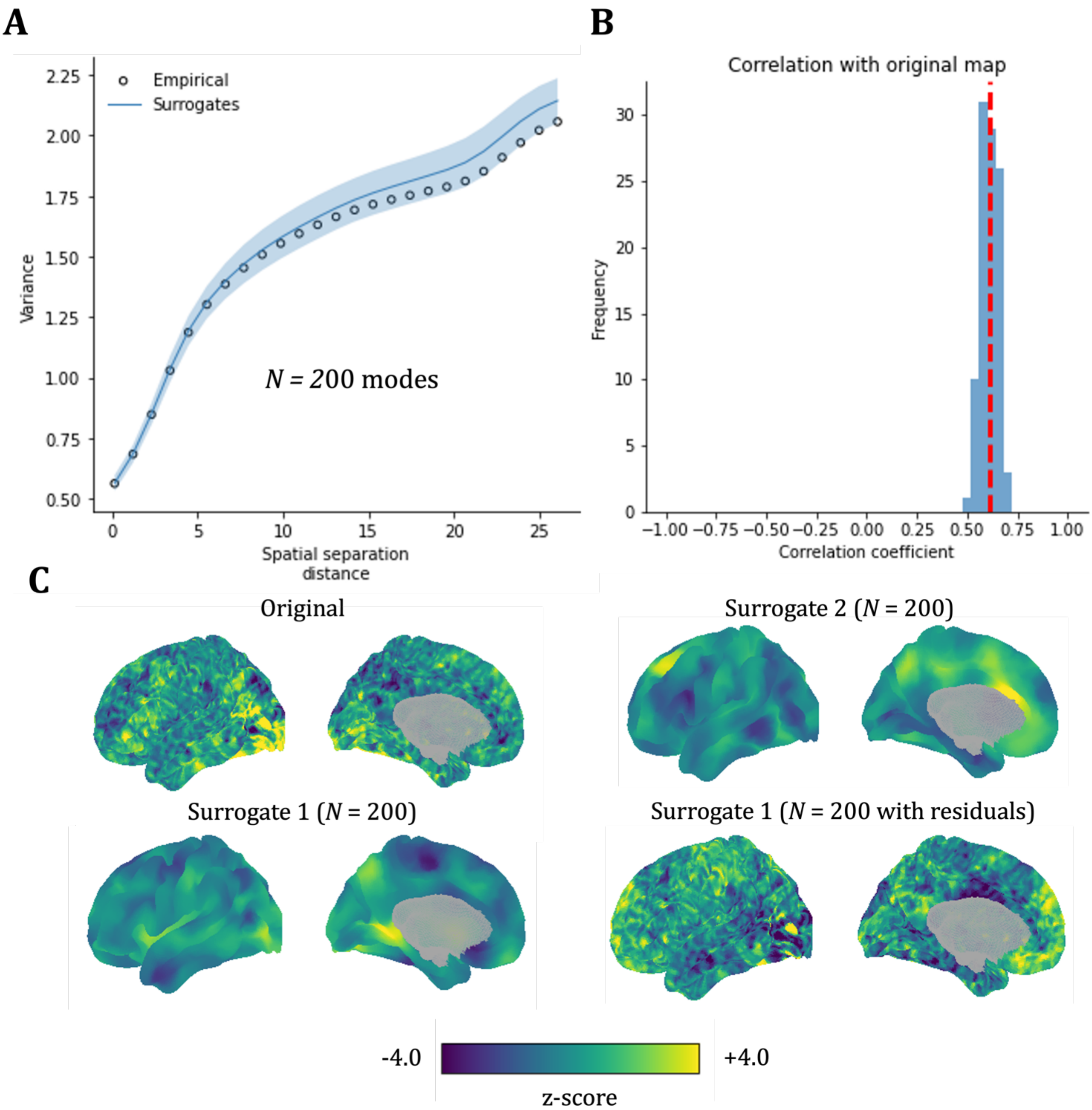

Fig. S5. The effect of including model residuals in surrogate brain maps. (A) Variogram of HCP task contrast data with N = 200 modes for modal decomposition. Note the closely matching variograms of the 1000 surrogates with original residuals added back to data after Eigenstrapping. (B) Including the original residuals in the surrogate distribution induces a strong correlation of the surrogates with each other and with the original data. Red dashed line is the average correlation of the surrogates with the empirical data. (C) Original map plotted alongside exemplar surrogates at N = 200. Notice the smoothness of the surrogates with low modes – these are then "recolored" by addition of the residuals to match the original, but this shifts the resulting distribution to highly correlated values unless the residuals are randomly permuted prior to inclusion.

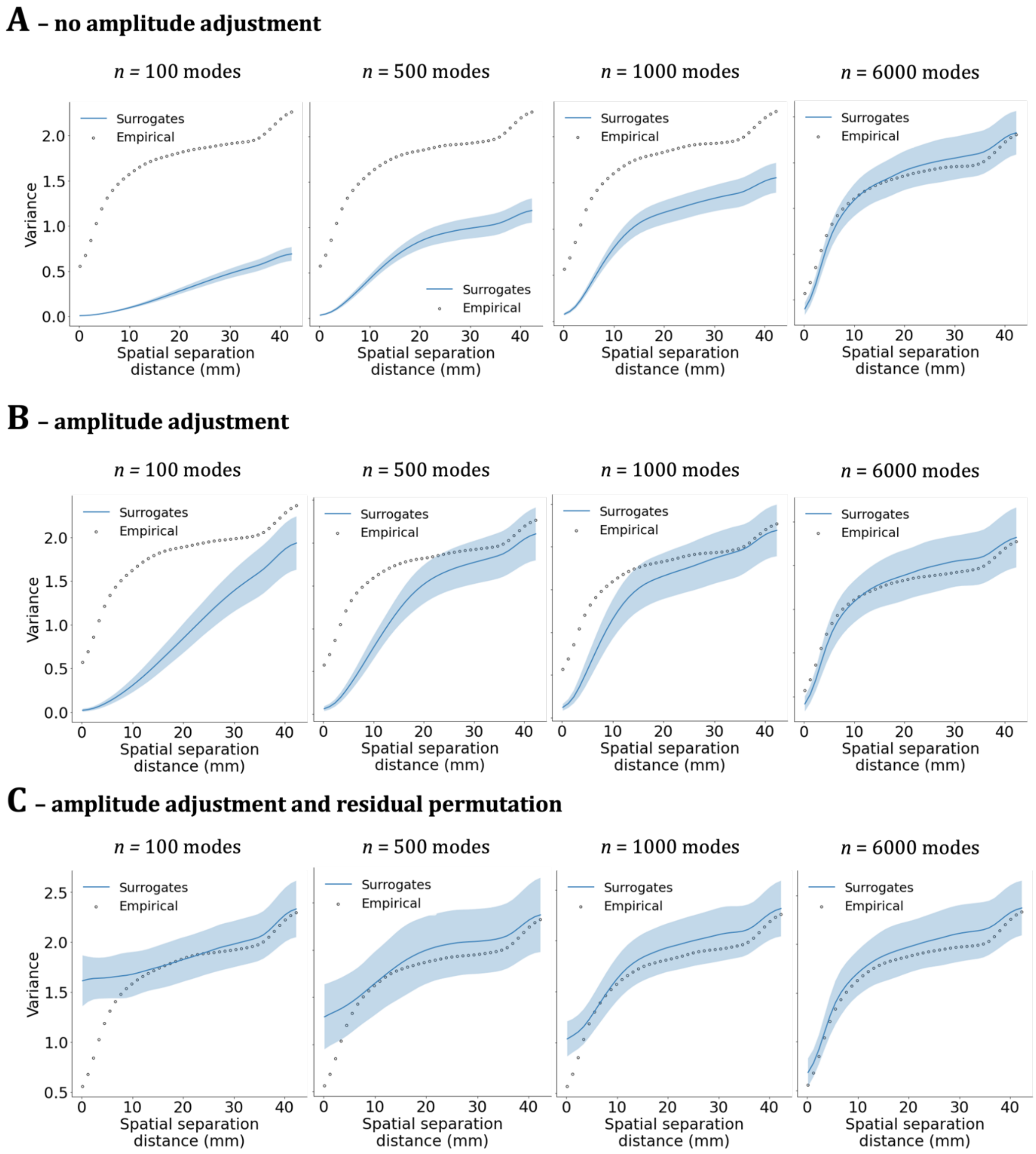

Fig. S6. Variograms of HCP *emotion* task data (see Supplementary Table 2) show the fit in (A) without amplitude adjustment is lower than in (B) where amplitude adjustment improves the fit of the surrogates (blue line) via a slight whitening effect, as well as broadening the standard deviation of the variograms (blue shaded area). Permuting the residuals increases the (zero-lag) variance of the surrogates in (C), which decreases to be in line with the empirical zero-lag as the number of modes increase.

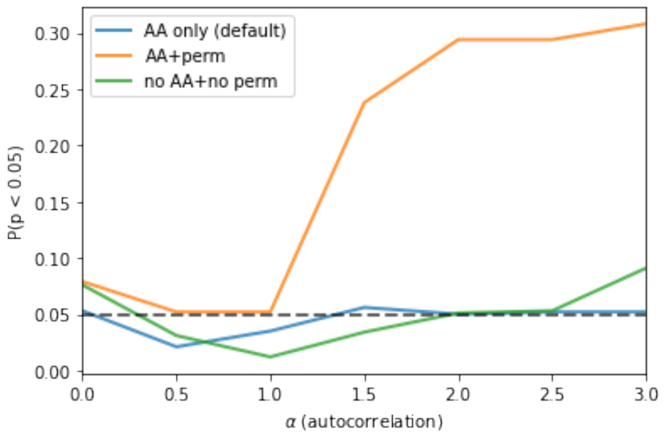

Fig. S7. Eigenstrapping false positives and the effect of amplitude adjustment (AA) and permutation of residuals. FPR with AA and no permutation of residuals (default; see Fig. 3) in blue; FPR with both AA and permutation in orange; FPR with no AA and no permutation in green.

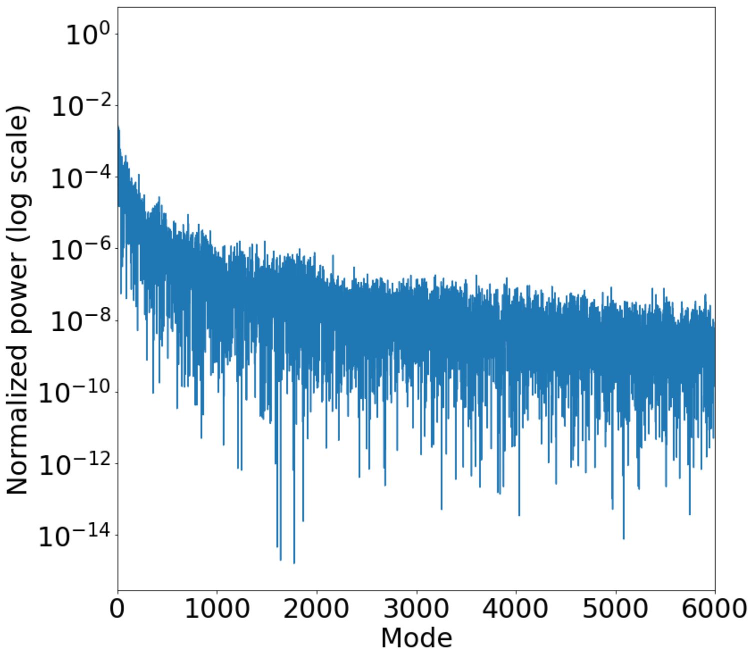

Fig. S8. Normalized eigenmode power spectral density derived from 6000 mode coefficients (Eq. 1; Fig. 1A).

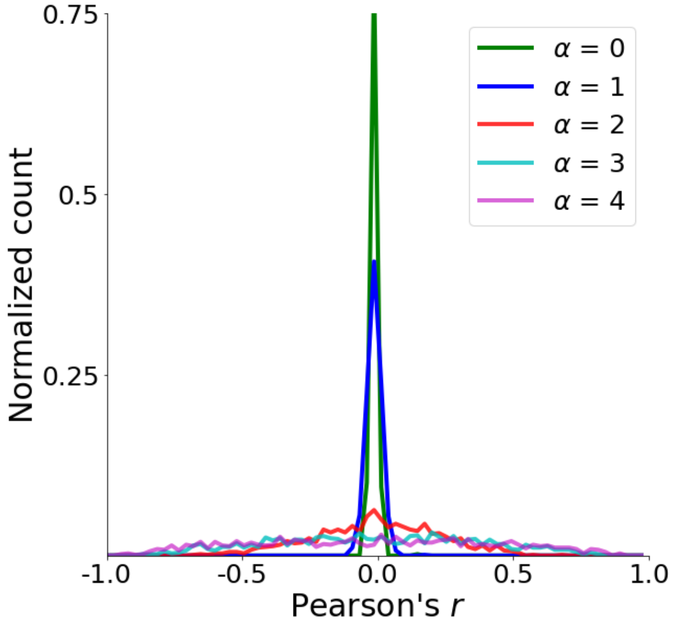

Fig. S9. Distribution of Pearson correlation between randomly paired generated maps as a function of α. Distributions are shown for α=0 (green), α=1 (blue), α=2 (orange), α=3 (cyan), and α=4 (magenta). Note that values are normalized by the total number of pairs for each α (1000).

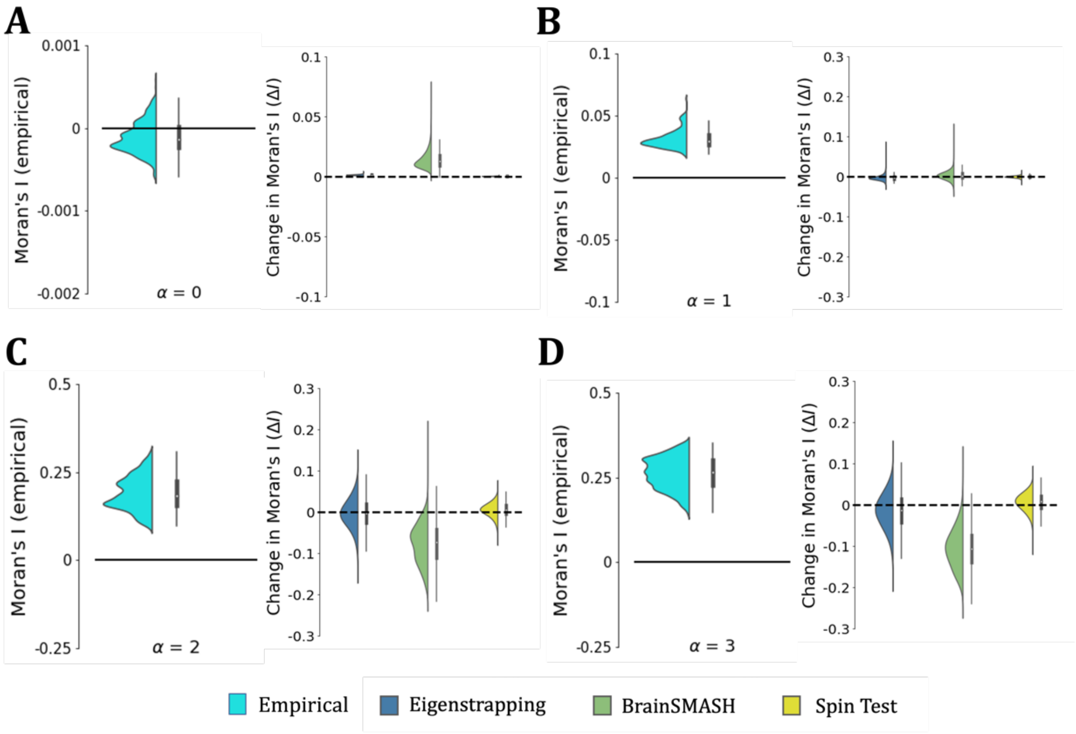

Fig. S10. Moran's I statistic computed on 1000 GRFs (left in light green) as a function of increasing SA. A: α = 0.0; B: α = 1.0; C: α = 2.0; D: α = 3.0. Change in Moran's I statistic (ΔI; empirical − null) is shown with rainclouds for eigenstrapping in blue, BrainSMASH in green, and Spin Test in yellow. Raw Moran's I values for empirical and surrogate classes for each α are given in Supplementary Fig. S6.

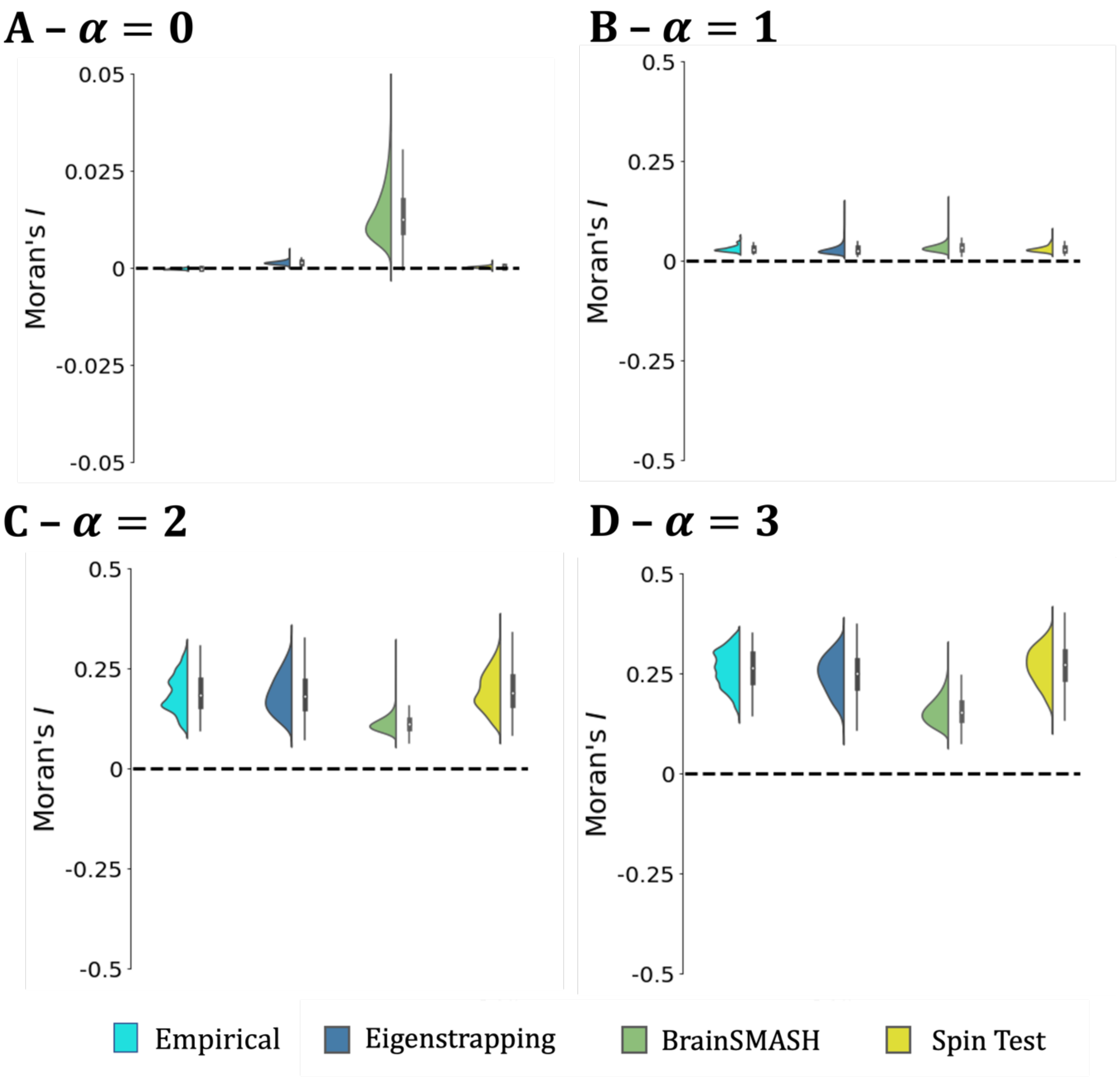

Fig. S11. Moran's I as a function of spatial autocorrelation α: (A) $\alpha = 0$, (B) $\alpha = 1$, (C) $\alpha = 2$, (D) $\alpha = 3$. Empirical values from simulated GRFs (1000 in each α) are plotted in cyan histograms; Moran's I of 1000 eigenstrapping surrogates of each GRF in blue; Moran's I of 1000 BrainSMASH surrogates of each GRF in green; Moran's I of 1000 Spin Test surrogates of each GRF in yellow

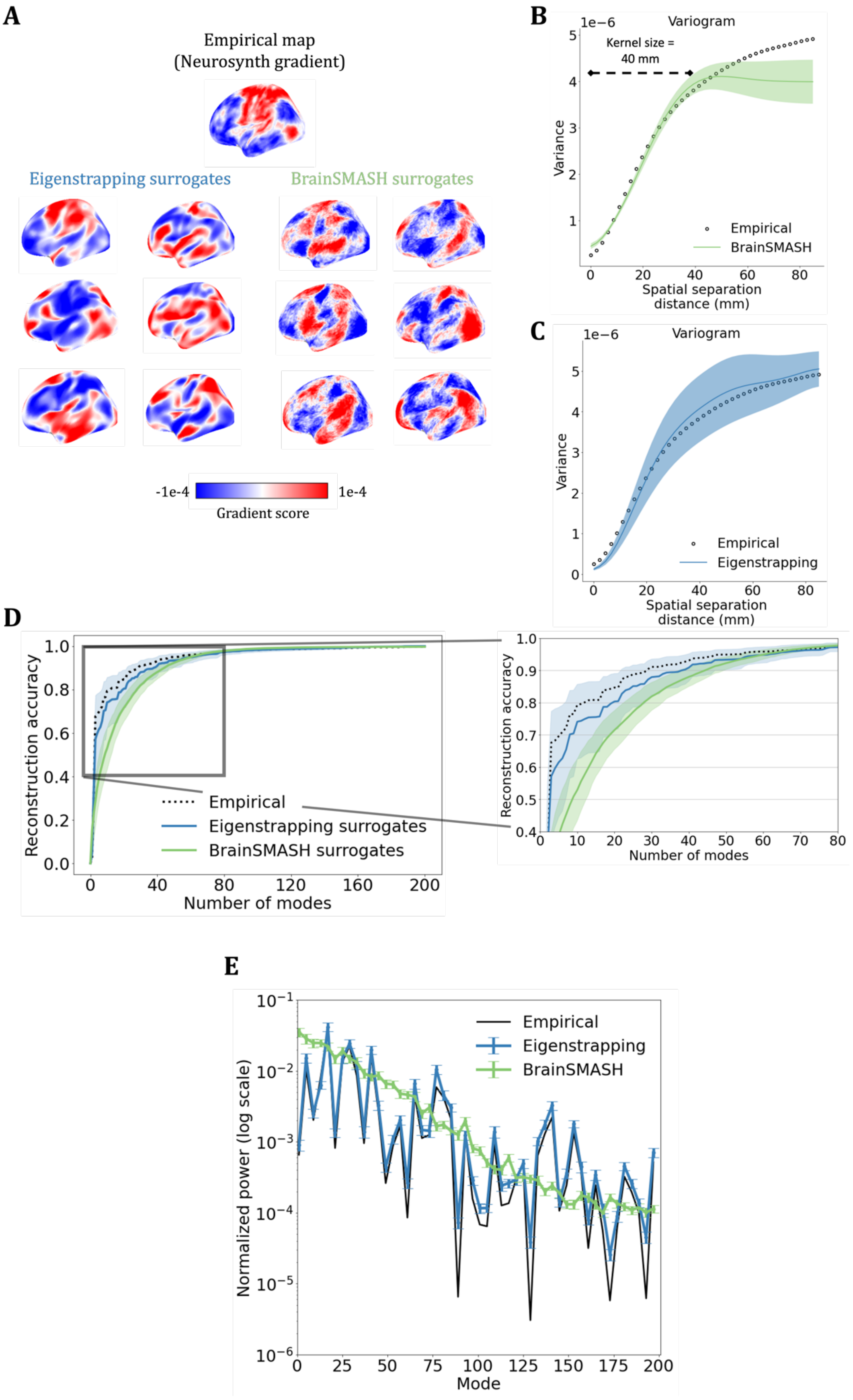

Fig. S12. Impact of BrainSMASH on spatial autocorrelation. (A) Empirical cognitive brain map (NeuroSynth cognitive terms 1st principal gradient) from Fig. 4 is shown above six

exemplar surrogates in each method. The whitening of the BrainSMASH surrogates is evident as the increased speckling. (B) The whitening effect is strongest at separations wider than BrainSMASH kernel (40 mm kernel). There also exists a slight whitening at the zero-lag variance (the intercept of the variogram). (C) Eigenstrapping replicates the variogram to very long ranges (>80 mm), with a greater variance than BrainSMASH at 40-60 mm. The mean variance of the surrogates follows the empirical curve across the entire spectrum. (D) Reconstruction of the empirical data (black dashed line), the eigenstrapped surrogates (blue), and BrainSMASH surrogates (green). The eigenstrapping surrogates show similar reconstruction to the original data. The inset panel shows the slower reconstruction accuracy of the BrainSMASH surrogates. (E) The average power spectrum of eigenstrapping surrogates (blue) is nearly identical to the empirical power spectrum (Pearson's r = 0.961). The average power spectrum of BrainSMASH surrogates (green) reproduces the slope, but not the variability of the empirical spectrum (Pearson's r = 0.401). Error bars in panel E denote standard error.

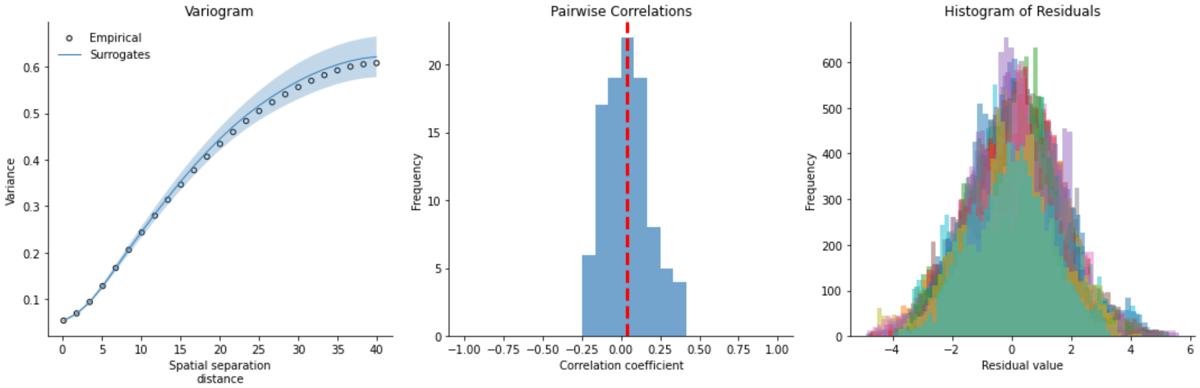

Fig. S13. Eigenstrapping diagnostic tools for the end-user. Left panel: Variogram of original data against surrogates. Middle panel: Pairwise correlations of surrogates with original data. Right panel: Histogram of residuals calculated from subtracting surrogates from original data.

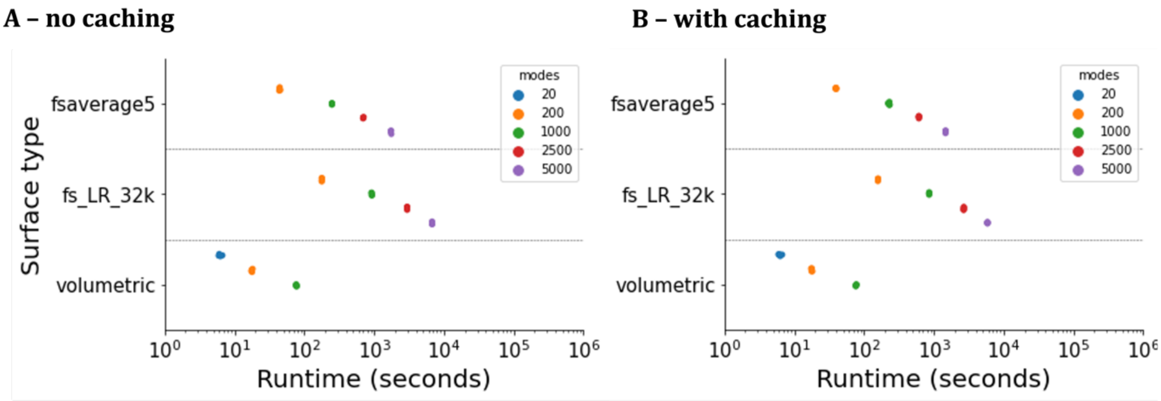

Fig. S14. Computation runtime for 1,000 surrogates using full algorithm (no pre-computed permutations). The computation time is plotted for *fsaverage* (top row), *fs-LR-32k* (middle row), and *volumetric* (bottom row) resolution data. Each null was run with different numbers of modes (20: blue; 200: orange; 1,000: green; 2,500: red; purple: 5,000), five times on a simulated brain map ($\alpha = 2.0$) for *fsaverage*; HCP task contrast data for *fs-LR-32k*; HCP cortico-subcortical gradient in the striatum for *volumetric*, consisting of 2,230 vertices). Repeats are plotted as separate dots. (A) The computation time of 1,000 surrogates with no pre-computation of modes. (B) The computation time of surrogates with pre-computation of modes. All computations were performed using one CPU/thread and without the "computation speed-up" with pre-computed permutation matrices (see Supplementary Information-S11). Specifications for the computational device are listed in Supplementary Table 3.

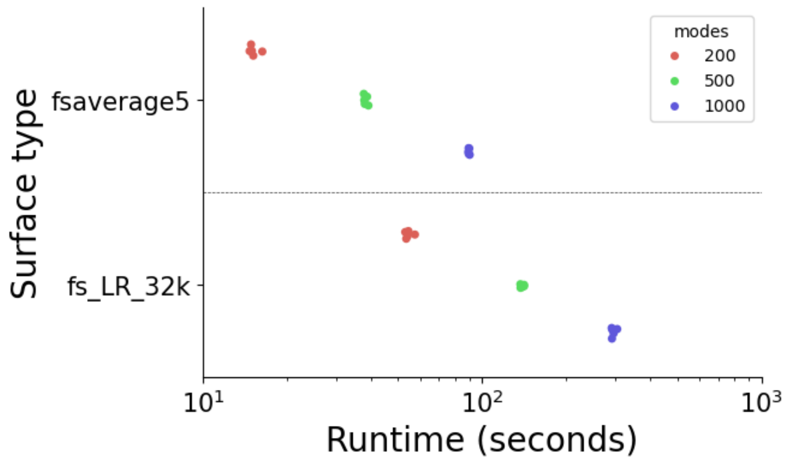

Fig. S15. Computation runtime for 1,000 surrogates with pre-computed permutation matrices. Computation time is plotted for the *fsaverage* (top row) and *fs-LR-32k* (bottom row) resolution data. Each null was computed with different numbers of modes (200: red; 500: green; 1,000: purple), five times on a simulated brain map ($\alpha = 2.0$) for *fsaverage* and HCP task contrast data for *fs-LR-32k*. Repeats are plotted as separate dots. All computations were performed using one CPU/thread. Specifications for the computational device are listed in Supplementary Table 3.

Supplementary Tables

Supplementary Table 1. Spatial wavelengths of the first 1024 eigenmodes.

**Eigengroup**	**Wavelength (mm)**	**Eigenmodes included in the eigengroup**
0	–	1
1	297.7	2–4
2	171.9	5–9
3	121.5	10–16
4	94.1	17–25
5	76.9	26–36
6	65.0	37–49
7	56.3	50–64
8	49.6	65–81
9	44.4	82–100
10	40.1	101–121
11	36.6	122–144
12	33.7	145–169
13	31.2	170–196
14	29.1	197–225
15	27.2	226-256
16	25.5	257-289
17	24.1	290-324
18	22.8	325-361
19	21.6	362-400
20	20.5	401-441
21	19.6	442-484
22	18.7	485-529
23	17.9	530-576
24	17.2	577-625
25	16.5	626-676
26	15.8	677-729
27	15.3	730-784
28	14.7	785-841
29	14.3	842-900

30	13.8	901-961
31	13.4	961-1024

Supplementary Table 2. HCP task contrasts.

Task type	Number of contrasts	Contrasts	Key contrast
social	3	random; tom; tom_random	tom_random
motor	13	cue; lf; lh; rf; rh; t; avg; lf_avg; lh_avg; rf_avg; rh_avg; t_avg; cue_avg	cue_avg
gambling	3	punish; reward; punish_reward	punish_reward
working memory (wm)	19	2bk_body; 2bk_face; 2bk_place; 2bk_tool; 0bk_body; 0bk_face; 0bk_place; 0bk_tool; 2bk; 0bk; body; face; place; tool; body_avg; face_avg; place_avg; tool_avg; 2bk_0bk	2bk_0bk
language	3	math; story; math_story	math_story
emotion	3	faces; shapes; faces_shapes	faces_shapes
relational	3	match; rel; match_rel	match_rel

Supplementary Table 3. Specifications for testing computer.

**Part**	**Hardware/Specification**
CPU*	2.4 GHz Quad-Core Intel Core i5
L2 Cache	256 KB (per core)
L3 Cache	6 MB
RAM	16 GB 2133 MHz DDR3
Graphics Card	Intel Iris Plus Graphics 655 1536 MB
OS	macOS Catalina 10.15.7

* note: for all results reported in Supplementary Information-S11 and Figures S15-S16, we did not perform any runs with more than one thread per run.
